# The Impact of Terroir on the Flavour of Single Malt Whisk(e)y New Make Spirit

**DOI:** 10.3390/foods10020443

**Published:** 2021-02-18

**Authors:** Maria Kyraleou, Dustin Herb, Grace O’Reilly, Neil Conway, Tom Bryan, Kieran N. Kilcawley

**Affiliations:** 1 Food Quality & Sensory Science Department, Teagasc Food Research Centre, Moorepark, Fermoy, P61 C996 Co Cork, Ireland; mkyraleou@yahoo.gr; 2 Crop and Soil Science Department Corvallis, Oregon State University, Corvallis, OR 97331, USA; herbd@oregonstate.edu; 3 Waterford Distillery, Waterford, Co Waterford, Ireland; grace@waterfordwhisky.com (G.O.); neilconway@waterfordwhisky.com (N.C.); 4 Boortmalt, Athy, Co Kildare, Ireland; tom.bryan@boortmalt.com

**Keywords:** terroir, GCMS, GCO, new make spirit, whisky, whiskey, barley variety, sensory evaluation, sensory panel

## Abstract

The impact of barley variety and its geographical growth location (environment) on the flavour of new make spirit was investigated to determine if “terroir” can be applied in the production of single malt whisk(e)y. New make spirits were produced at laboratory scale under controlled conditions from two different barley varieties (Olympus and Laureate) grown at two distinct environments (Athy, Co Kildare and Bunclody, Co Wexford) in Ireland over two consecutive seasons (2017 and 2018). The spirit samples were analysed by gas chromatography mass spectrometry olfactometry and descriptive sensory analysis. Forty-two volatiles were detected with eight deemed as very influential and fifteen deemed as influential to the aroma of new make spirit. Sensory attributes were influenced by barley variety, environment, and the interactions thereof over both seasons, with environment and the interaction of variety x environment having a greater impact than variety alone. Chemometric analysis of the olfactometry and sensory data found that both environment and season had a greater impact on the aromatic sensory perception of the new make spirits than variety alone. Therefore, this study clearly demonstrates a “terroir” impact on the flavour of new make spirit and highlights its potential importance especially in relation to single malt whisk(e)y.

## 1. Introduction

The flavour of single malt whisk(e)y depends on the type of grain, malting, mashing, distilling, and maturation processes used. The new make spirit, which describes the product post distillation and prior to cask maturation, must be stored in wooden casks for at least 3 years [1] before bottling and retail.

The new make spirit contains a high number of compounds, the most abundant being esters and alcohols. Esters are produced mainly during alcoholic fermentation by yeasts and contribute to fruity (e.g., ethyl hexanoate, isoamyl acetate) and floral aromas (e.g., 2-phenylethyl acetate). Alcohols originate from the raw material and are formed after harvest and during the malting process from lipids in the presence of oxygen through a sequence of enzymatic reactions known as the lipoxygenase pathway (e.g., hexanol) [2,3,4]. Other alcohols are also produced during fermentation (e.g., 2-methyl-1-butanol, 3-methyl-1-butanol) by yeast through decarboxylation and the reduction of α-keto acids to their corresponding aldehydes and then via reduction to alcohols [5]. Other chemical groups that contribute to the overall aroma of new make spirits are ketones, aldehydes, terpenes, sulphur, and furan compounds [6,7,8,9,10]. The flavour of mature whisk(e)y is also impacted by a series of reactions occurring during the maturation process in the cask, but these are not explored in this study.

Profiling the volatiles in a product provides information on the evolution of flavour compounds that potentially influence sensory perception. The standard approach involves extraction and concentration of the volatiles prior to separation and identification (qualitative or quantitative) by gas chromatography mass spectrometry (GCMS). However, as it is estimated that only a small portion of volatile compounds actually contribute to sensory perception [11], therefore, it is beneficial to use gas chromatography oflactometry (GCO) to determine those volatiles that are actually influencing sensory perception. Basically, GCO uses trained assessors to sniff the eluent from the GC column containing the separated volatiles. In effect, it uses the human nose as an additional detector, as the eluent is typically split between a mass spectrometer and/or a flame ionisation detector. In this way, individual volatiles can be identified and their aromas classified in real time. The aromatic contribution of a volatile compound to the overall aroma of a sample can be estimated by a range of different GCO methods [10,12,13,14,15]. Poisson and Schieberle determined that 26 key odorants were responsible for Bourbon aroma out of a total of 45 volatile compounds detected [15,16]. In another study, 34 out of 150 volatile compounds were deemed responsible for the characteristic odour of freshly distilled Cognac [17].

Although the flavour of aged and new make spirits are quite different, the most important feature of both is their volatile aromatic profile, which originates from the raw materials used, processing practices, and conditions. The concentrations of individual volatile compounds in new make spirit and their contribution to the total aroma were found to be associated to the botanical origin of the raw material [18,19]. Recently, work by Herb et al. found that the cereal crop imparted a distinctive sensory profile to beer, which was also directly attributable to its geographical origin and thus associated environmental and soil conditions [20]. Additionally, an environmental effect on the volatile profile of the new make bourbon was recently proposed [9]. This concept is well established under the term “terroir” in the wine industry [21], but it has not been significantly studied in other alcoholic beverages. Terroir is described as the set of all environmental factors that affect a crop’s phenotype, including unique environmental contexts, farming practices, and the crop’s specific growth habitat. It represents the quality and the unique sensory characteristics of wine as it is directly connected to the geographical origin of the product. Numerous studies have revealed the correlation of geographical origin of an agricultural product with its quality, and the authors expressed the need for many more studies to further establish the terroir concept in other products [22]. The contribution of terroir in whisk(e)y production has not been studied mainly due to the fact that the flavour synthesis during production, including maturation, is thought to overwhelm or diminish any direct impact of the raw materials. In addition, most whisk(e)y producers pool malted barley from different varieties and from barley grown at different locations, and therefore, the resultant new make and aged spirits are an amalgamation of multiple raw materials, their associated factors making it impossible to discern any potential “terroir” effect. However, producing new make spirit from the same barley varieties grown at independent geographical locations selected on the basis of growing on different environments and processed separately can be used to determine potential “terroir” effects.

Therefore, this study was undertaken to determine if the volatile and sensory profile of new make spirit was influenced by barley variety and environment with all processing carried out under controlled conditions. The study was undertaken over two seasons, and the environments of the barley plots were chosen based on distinct soil types and weather patterns.

## 2. Methods

### 2.1. Chemicals

Ethyl hexanoate, ethyl decanoate, 1-octen-3-one, (E)-2-nonenal, (E)-2-octenal, hexanal, heptanal, methional, β-damascenone, ethyl 3-methylbutyrate, ethyl isobutyrate, 3-methylbutyl acetate, 3-methyl-1-butanol, 1,1-diethoxyethane, 3-methylbutanal, 2-phenylethanol, 2-phenylethyl acetate, 2-pentylfuran, and 2-ethylfuran were purchased from Merck, Ireland (Arklow, Co., Wicklow, Ireland) and ethanol absolute (Scharlab, S.L., Barcelona, Spain).

### 2.2. Experimental Conditions

Barley from the varieties Olympus (LGB 11-8339) and Laureate (SY 412-328) were grown at two distinct environments (Athy, Co Kildare and Bunclody, Co Wexford) in Ireland and harvested over two consecutive seasons (2017 and 2018). Field soil associations were identified from the Teagasc soil maps (http://gis.teagasc.ie/soils/): Athy (2017 52.9896, −6.9944 and 2018 53.0387, −7.2128 Coordinates)—Elton Association—fine loamy drift with limestones; Bunclody (2017 52.634, −6.6092 and 2018 52.5808, −6.5922 Coordinates)—Clonroche Association—fine loamy drift with siliceous stones. Soil samples from those two trial locations were analysed in accordance with national guidance [23] (IAS Laboratories, Bagenalstown, Co Carlow, Ireland). Soil nutrients and quality can be viewed in Supplementary Table S1. Athy soil is alkaline (high pH) indicated by substantially higher levels of Ca, Mg, and Mo and lower levels of Fe, Cu, Mn, and B compared to Bunclody. Additionally, P was greater in Athy, which is possibly linked to an increased prevalence of Fe, Al, and Mn hydroxides associated with high pH (Supplementary Table S1). Weather patterns varied across environment and seasons: Athy, being more inland, had more consistent higher temperatures and lower rainfall during the growing season in comparison to Bunclody, which is more coastal and subjected to more volatile weather patterns (Supplementary Table S2). Across both environments, 2017 consisted of cooler average temperatures and higher rainfall throughout the growing season, whereas 2018 was warmer and dryer in comparison with higher average temperatures and lower annual precipitation (Supplementary Table S2).

Both trials were sowed in a randomised complete block design (RBCD) on 19th–22nd of March and 27th–30th of April in the 2017 and 2018 seasons, respectively. In the 2017 season, the barley crops were harvested on 15th and 31st of August in Bunclody and in Athy, respectively, while in 2018, both crops were harvested on 8th–10th of August. Sowing and harvesting of the crops as well as micro-malting of the barley were undertaken by Minch Malt Ltd. (The Maltings, Athy, Co Kildare, Ireland). Samples (16 kg each) were micro-malted following the malting protocol steeping, 5 h wet at 15 °C, 15 h couch at 15 °C, 6 h wet at 15 °C, 15 h couch at 15 °C; germination, 24 h at 16 °C with 30% air flow and 50% recirculation and a temperature ramp of 1 °C per 24 h until 13 °C; and kilning, 6 h at 58 °C, 5 h at 63 °C, 2 h at 68 °C, 6 h at 72 °C, and 2 h at 71 °C with 50% air flow and 40% recirculation.

### 2.3. New Make Spirit Production

Mashing and distillation of the malted barley were undertaken by Tatlock and Thomson Ltd. (Tatlock House, By Leven, Fife, Scotland) according to the following procedure. Approximately 10 kg of malt for each sample was ground in a laboratory 3-roller mill. The malt grist was mashed with 28 L of hot water at 65 °C and then with 14 L of hot water at 85 °C into a laboratory mash tun. The water from each mashing was drained, combined, and approximately 28 L of wort was collected. The wort was fermented using Anchor Dried Distilling Yeast (Lallemand Dried Malt Distillers Yeast, Johannesburg South Africa) for 72 h at 32.5 °C. Following fermentation, the wash was distilled in a laboratory spirit still for the collection of new make spirit of alcohol content not more than 76% alcohol by volume (%ABV).

### 2.4. Gas Chromatography Olfactometry (GCO) Method

The equipment for the analysis of the samples is located in a dedicated laboratory with temperature control where trained panelists could sniff free of distraction. For every session, a diluted new make spirit sample of 5 mL (20%ABV) was added to a 20 mL amber La-Pha-Pack headspace screw capped vial with a magnetic cap and silicone/polytetrafluoroethylene septa (Apex Scientific, Maynooth, Co. Kildare, W23 R1H2, Ireland). A Divinylbenzene/Carboxen/Polydimethylsiloxane (DVB/CAR/PDMS) solid phase micro-extraction (SPME) fiber (Agilent Technologies Ireland Ltd., Cork, T45 YX04, Ireland) in an MPS Gerstal autosampler (Anatune Ltd., Cambridge, UK) was exposed to the headspace above the sample at 40 °C for 40 min with agitation using the heater/agitator module. Analyses were performed on an Agilent 7890 Gas Chromatography System using a DB-624UI mid-polar column (20 m × 0.18 mm × 1.0 µm, Agilent Technologies Ireland Ltd, Ireland.) equipped with an Agilent 5975C inert XL mass selective detector (MSD), a flame ionisation detector (FID) and a Gerstal Olfactory Detection Port (ODP 3) (Anatune Ltd., Cambridge, CB3 0GP, UK). The carrier gas was helium, held at a constant flow of 1.2 mL/min, and a 3-way splitter was used to split the flow proportionally (1:1:1) from the analytical column to the three detectors (MSD, FID and ODP 3. The transfer lines (deactivated silica capillary tubing) from the splitter were measured between the MSD (3.3 m × 0.15 mm), FID (2.14 m × 0.15 mm), and the ODP3 (2.14 m × 0.15 mm) to ensure peak alignment. The total GC run time was 29 min. The initial oven temperature was 40 °C for 2 min, raised to 140 °C at 6 °C/min, and finally to 220 °C at 15 °C/min and held for 5 min. Detection was performed in full scan mode with the mass range between 35 and 250 amu. All samples were analysed in triplicate. Sniffing was carried out in a temperature controlled room (21 °C), and humidified nitrogen was added to the transfer line to prevent nasal mucosa dehydration. The compounds were identified by olfactory descriptions, reference analytical standards, and/or mass spectra comparison to the NIST 2014 mass spectral library and their linear retention indices (LRI) were calculated using homologous series of C5–C20 n-alkanes.

### 2.5. Panel Training for GCO Sessions

During training, potential panelists were screened to determine their individual olfactory sensitivity using reference standards and their ability to recall and recognise an odour as described in Barttoli et al. and Vene et al. [12,24].

Panelists became familiar with the GCO equipment and their ability to smell and identify the presence of an odour in random samples over 10 independent sessions. At the 1st session, they were screened for their ability to recognise an odour using the Test Blue Kit with 16 Sniffin’ Sticks (Burghart Messtechnik GmbH, Wedel, Germany). During the screening test, it was observed that some panelists described the odours differently. For this reason, a vocabulary adaption (Supplementary Table S3) was proposed as used by Konstantinidis et al. [25]. Each pen (distinct odour) was presented once, and panelists were asked to make a choice from a list of 4 possible descriptors for each of the 16 pens (odours). Those who could identify more than 12 out of 16 odours passed this initial screening test. In the meantime, a 3-member expert panel was asked to sniff 5 different spirit samples and note the retention times and the attributes of the detected odours. The results were evaluated in order to create a table containing all the detected odours (Supplementary Table S4), which was used by panelists for the characterisation of the samples. The odour zones have also been identified, and the sniffing time was determined from 3 to 25 min. At the next three sessions (2nd, 3rd, 4th session), a selection of relevant reference standards (Supplementary Table S5) were given to the screened panelists, and a vocabulary was also developed. The reference standards were compounds or odours that are characteristic of new make spirits and were selected by the 3-member expert panel. The vocabulary was presented and then intensity assessment using a scale (0–3) was undertaken, 0 = not detected, 1 = weak odour, 2 = clear, but not intense odour, and 3 = very intense.

In the next three sessions (5th, 6th, 7th session), panelists were trained in GCO analysis with the reference standard mix (Supplementary Table S5). They were asked to describe the odour and rate the odour intensity using the 0–3 scale (as described earlier); however, half values were accepted if panelists felt they could not ascribe 0, 1, 2, or 3. On the 8th session, the actual new make spirit samples were presented to the panel via the GCO and each panelist was asked to describe the odour with their own choice of words. In the final sessions (9th and 10th session), each panelist was trained in GCO analysis with a new make spirit sample. At this point (Supplementary Table S4), they could detect the odours, circle the most appropriate characterisation of an odour, and rate the perceived intensity of each compound on the point intensity scale using numbers (half numbers were accepted).

From a total of 9 volunteers, 6 panelists were selected that met all the criteria.

### 2.6. Olfactory Tests in GCO

Olfactory tests of the new make spirit samples were carried out by six trained panelists (four females and two males). They were asked to sniff through the ODP 3 olfactory port for 22 min per run and to record the intensity and describe the odour as per training. All samples were analysed in triplicate using a splitless mode injection, and the odour intensity of each aroma was determined as the average value of the results from all panelists. The modified frequency percentage (MF%), which indicates the most important odorous compounds, was calculated using the following formula:

$$\mathrm{MF}\left(\%\right)=\sqrt{F\left(\%\right)\times I\left(\%\right)}\;$$

where F (%) is the detection frequency of an aromatic attribute expressed as percentage. I (%) is the average intensity expressed as a percentage of the maximum intensity. The most important compounds are those with an MF > 50% [12].

In addition, aroma extraction dilution analysis was also determined by adjusting the GC injector split ratio [26] in order to estimate the importance of the detected compound and eliminate any adverse sample matrix effects by diluting the sample. The volume of the sample was 5 mL of diluted spirit (20% ABV), the split ratio was adjusted to 5:1, 10:1, 20:1, 40:1, 80:1, and 160:1, and each sample dilution was analysed twice until no odour was detected. Panelists had to detect the odour at each session without evaluating the intensity. The flavour dilution factor (FD) of the volatiles was calculated as the mean values of FD from each panelist, which represented the split ratio (5, 10, 20, 40, 80, and 160). The FD of an odour detected at the splitless injection was 1, and thus, the potential highest or maximum FD achievable was 160.

### 2.7. Sensory Evaluation of New Make Spirits

Descriptive sensory analysis of new make spirits was undertaken by Tatlock and Thomson Ltd. (Fife, Scotland) with a trained panel consisting of six panelists according to the general guidance described by ISO 13299:2016 method for sensory analysis [27]. Fifteen sensory attributes (pungent, feinty, cereal (grain), malty/biscuity, green/grassy, floral, fresh fruit, dried fruit, soapy, solventy, sweet, oily, sour, sulphury, stale/mouldy) were used for the evaluation of the spirit samples, and panelists were asked to rate the intensity of each attribute based on a scale from 0 to 5, where 0 was not detectable and 5 was the most intense. Due to the high ABV of new make spirit, only two experimental samples and an internal standard were assessed per session in order to avoid panelist fatigue. The descriptive sensory analysis was carried out using Glencairn glasses in a controlled environment. All panelists had extensive experience in the descriptive analysis of spirits including whisk(e)y and new make spirits.

### 2.8. Statistical Analysis

Statistical analyses were conducted with JMP Pro statistical software (version 14, SAS Institute, Cary, NC, USA). A mixed linear model approach was used to analyse the sensory data. Analyses of variance (ANOVAs) were performed for the sensory attributes. Student’s t-test was used to calculate F-protected least significant differences for mean separation, and the Bonferroni correction was applied to adjust the critical *p*-values to reduce the incidence of false positives. Statistical analysis of MF% values of aromatic compounds was submitted to a variance analysis (ANOVA), on Statistica V.7 Software (Statsoft, Inc., Tulsa, OK, 74104 USA). Comparison of mean values were performed using Tukey’s HSD (honestly significant difference) test when samples were significantly different after ANOVA (*p* < 0.05). Pearson’s correlation analysis was used to investigate relationships between sensory attributes and MF% values of aromatic compounds with SPSS.

## 3. Results

### 3.1. Aroma Active Compounds

Forty-two odours were detected by olfactometric analysis, and 32 of them were fully or tentatively (denoted as*) identified by their olfactory descriptions, reference analytical standards, and/or a mass spectral library. These consisted of the following: 2 acetals, 4 alcohols, 10 aldehydes, 10 esters, 2 ketones, and 4 furans. Ten unidentified compounds were also included with odour descriptors (Table 1). This is not uncommon in GCO analysis and highlights that a volatile compound was present above its odour threshold but below its limit of detection, or that it was co-eluting with other compounds, and we could not get a sufficiently independent MS profile for identification purposes. As the objective of panel training was to assign an odour descriptor relevant to the compound but familiar to all the panelists in order to have standardised results, the descriptors in this study may be different to those presented in previous studies. For example, we described β-damascenone as honey/tea/plum, while it has been previously described as cooked apple [15] or peach/canned apple [28], although it was first isolated from roses (Rosa Damascena) and was characterised as “rose ketone”. As this compound has been detected in a wide range of foods, its sensorial description has also been linked to product type [29]. High variability in odour descriptors was also observed for (E)-2-nonenal; we described it as fried/toasted/fatty but it has previously been described as green [15] or cardboard, leather, or shoe box [30].

The identification of (E)-2-octenal, (E)-2-nonenal, decanal, methional, and 1-octen-3-one was difficult due to their low abundance, which resulted in a weak mass profile. We selected potential standards based on ion profiles, retention index, and odour characteristics. Identification was confirmed through trial and error by spiking potential standards in a new make spirit sample and reassessing as described earlier. In contrast, ethyl octanoate, ethyl decanoate, and ethyl dodecanoate, which had high peak abundances, were easily identified through the mass spectral profile and retention index, but these volatiles had very low odour impacts, and in many cases, they could not be perceived by the trained panel (Supplementary Figure S1). These were not included in Table 1, as it was concluded that they had no or a minimal odour impact; however, they may indirectly contribute by synergistic or masking interactions. The detected odours were grouped into eight distinct categories according to their aroma (chemical, earthy, fatty, floral, fruity, grassy, vegetal, and roasty) in order to more easily discuss how their individual MF% was impacted by barley variety, environment, and season (Table 1 and Table 2). The highest number of volatile compounds that contribute to the new make spirits aromatic profile (MF > 50%) belong to the earthy and fruity categories, while the individual compounds with the highest MF values and thus the most intense were fatty and grassy aromas.

#### 3.1.1. Chemical Odours

The compounds with chemical aromas—nonanal (soap/fresh), ethyl acetate (glue/nail polish remover), and 2-ethylfuran (plastic)—had low average MF values (42.4%, 12%, and 12%, respectively), and thus are less likely to have a major contribution to the aroma of new make spirits (Table 1). None of the MF% values of these compounds were impacted by barley variety, environment, or season.

#### 3.1.2. Earthy odours

The most aroma active earthy odours based on average MF% were 1-octen-3-one, 2-pentylfuran, and two unidentified compounds (unknown 1 and unknown 5) (Table 1). The MF% values of 1-octen-3-one (metallic/mineral/mushroom) (average MF 72.6%) were impacted by the season (higher in 2018). 1-Octen-3-one has been previously detected in freshly distilled cognac [17]. The MF values of 2-pentylfuran were impacted by season and environment (higher in 2017 and at Athy) with an average MF value of 56.6% (Table 1 and Table 2). The odour of 2-pentylfuran (gas/bad smell) has been shown to differ depending upon its abundance; it was previously characterised as nutty at low abundance but pungent at high abundance [4]. 2-Pentylfuran has been previously reported in new make bourbon [9] and in barley during the malting process [4].

The unknown compound 5 had a soil herbal aroma and an average MF of 61.9% and was impacted by season and environment (higher in 2017 and at Bunclody). The other unknown compound (unknown1) (soil) with an average MF value of 52.1% was impacted by season (highest in 2017). 2-Butylfuran (stable) had the lowest average MF within the earthy aromas category at 30.4% and therefore of low sensory importance. It was impacted by season and environment (highest in 2018 and at Athy) and is uncommon in distilled spirits (Table 1 and Table 2).

#### 3.1.3. Fatty Odours

Fatty aromas in these samples were due to aldehydes and alcohols (Table 1). The most important compounds in the category were (E)-2-nonenal (fried/toasted/fatty), 3-methyl-1-butanol (fermented/yeast/rancid), and 3-methylbutanal (butter/cheese/chocolate). (E)-2-Nonenal is generated through lipid oxidation of linoleic and linolenic acid during the malting process [4,15] and from wood during maturation [31], but it has also been detected at trace levels in barley [4,32]. (E)-2-Nonenal was found to contribute to the green notes of Scotch malt whisky [33], and it was identified as a significant compound for new make and aged Bourbon aroma [9,16]. However, Arnold et al. (2019) has also linked high concentrations of (E)-2-nonenal to unpleasant aromas [9]. The high average MF values for these three compounds (E)-2-nonenal, 3-methyl-1-butanol and 3-methylbutanal at 94.2%, 87.1% and 60%, respectively reflect their significant importance to the aroma of new make spirits (Table 1). The MF% (E)-2-nonenal intensity was impacted by environment (higher at Athy) (Table 1 and Table 2). The chemical pathways for the formation of 3-methylbutanal and 3-methyl-1-butanol are similar [5]. 3-Methylbutanal is derived from the amino acid leucine and is a characteristic volatile compound in some dessert wines [34]. It is considered as an active odour compound of brewing barley [35] and of aged bourbon [15], where the odour was described as malty. Dong et al. (2013) [4] observed that the malting and fermentation processes impacted on aldehydes and that a significant formation of 3-methylbutanal occurred during the roasting process. The MF% of 3-methylbutanal was not impacted by season, variety, or environment. The alcohol 3-methyl-1-butanol has a lower odour threshold compared to its precursor (3-methyl-butanal) and is frequently found in alcoholic beverages [7,16,17,36,37]. The concentration of 3-methyl-1-butanol in relation to other major volatile compounds has being proposed for the authentication of Scotch and Irish whisk(e)y [38]. It is mainly produced during fermentation and is affected by the fermentation conditions [39]. At high concentrations, 3-methyl-1-butanol can confer an undesirable aroma, but it has been described as burnt/whisk(e)y [40], malty/rancid/pungent [26], plastic [6], and chocolate [17]. 3-Methyl-1-butanol was described as having a fermented/yeast/rancid odour in this study, but MF% was not impacted by season, variety, or environment.

2-Methylbutanol (rancid/buttermilk) had an average MF of 47.9%, and it was not influenced by season, variety, or environment. 2-Methyl butanol content has been previously used with 3-methyl-butanol to authenticate Scotch whisky [41]. 2-Methyl-1-propanol (butter/baked) is also an important volatile contributing to the flavour of whisk(e)y; it is formed during fermentation, and its production can be affected by the choice of yeast strain [42]. 2-Methylpropanol also known as isobutanol had an average MF value of 40.9% and was not impacted by season, variety, or environment. 2-Methylpropanal (butter/rancid) also known as isobutyraldehyde had a much lower average MF at 16.8% and therefore of much less importance and was not influenced by environment, variety, or season (Table 1 and Table 2). 2-Methylpropanal is predominately produced during fermentation and has been associated with unpleasant tastes in spirits [43].

#### 3.1.4. Floral Odours

Five volatile compounds were grouped in this category (Table 1). β-Damascenone was one of the most important floral odours detected in these new make spirits and has previously been described as a key aroma compound in aged bourbon [15]. It was characterised as an important contributor to the aroma of alcoholic beverages with a high odour activity and a low odour threshold [16,44,45]. In wines, it was reported that β-damascenone interacts synergistically with other compounds to enhance fruity aromas [44,45] or can also minimise negative vegetal aromas [28,45], which further highlights its potential significance to the aroma of new make spirit and whisk(e)y. The average MF values of β-damascenone in the new make spirits was 82.3% and was influenced by environment (higher at Bunclody) (Table 1 and Table 2).

Another very important floral odour based on its average MF value (77.9%) was 2-phenylethanol, which has a rose/floral aroma. 2-Phenylethanol is produced during fermentation, and the interaction with acetic acid leads to the formation of 2-phenylethyl acetate. 2-Phenylethyl acetate (floral) had an average MF of 58.8%, and it has been previously described as giving a roses/flowery/honey nuance to whisk(e)y [7]. However, the MF% values of both compounds were not impacted by season, variety, or environment, but it has been shown to be an important aromatic component of different whisk(e)y, cognac, and calvados [6,7,17]. An unknown compound (Unknown compound 4) (floral/fruity) had an average MF of 48.8% and was influenced by season (higher in 2017) (Table 1 and Table 2). 2-Phenylacetaldehyde (or benzeneacetaldehyde), which was described as floral aroma, had a low average MF at 26.6% and was therefore unlikely to significantly contribute to the aroma of new make spirit. It has been previously detected in barley as an important odour-active compound and was correlated with barley variety [35]. The MF% values of 2-phenylacetaldehyde were influenced by season and variety (higher in 2018 and Olympus) (Table 1 and Table 2).

#### 3.1.5. Fruity Odours

Eleven volatiles were grouped in this category (Table 1). The fruity aromas of new make spirits were due to esters and some acetals, and the most representative compounds of these categories were ethyl hexanoate, which is an unknown compound (unknown compound 8), 1,1-diethoxy-2-methyl-propane* (tentatively identified), ethyl isobutyrate, 3-methylbutyl acetate (also known as isoamyl acetate), 1,1-diethoxyethane, ethyl butyrate, and ethyl-3-methylbutyrate as their average MF was >50%.

The average MF value of ethyl hexanoate (pear/fruity) was 84.9%, indicating its significant contribution to the aroma of new make spirit, and it was not impacted by season, variety, or environment (Table 1 and Table 2). Ethyl hexanoate is a fatty acid ethyl ester produced during fermentation by a combination of ethanol and hexanoic acid, and it has been previously identified as a major aromatic compound of new make and aged bourbon [9,16], and an important compound in other alcoholic beverages [6,17,46]. In red wines, ethyl hexanoate confers a red-berry aroma [47] and is known to enhance fruity notes in grape spirits [17]. The unknown compound 8 also had a high average MF at 84.2%, indicating its potential significance to the aroma of new make spirit. Unknown compound 8 has the aroma of lemon/cleaning fluid, and its MF% was influenced by season (higher in 2018). Another ester that contributed to the fruity aroma of the new make spirit samples was 3-methylbutyl acetate (isoamyl acetate) with the characteristic odour of banana/fruity/melon with an average MF of 70.4%. The MF% was not impacted by season, variety, or environment. It has been previously identified in distillates [6,15,17] and is produced from a combination of 3-methyl-1-butanol and acetic acid. Ethyl isobutyrate (also known as ethyl 2-methylpropanoate) (fruity/sweet/cherry) formed from ethanol and isobutyric acid (2-methylpropanoic acid) had an average MF of 62.7% and was influenced by season (higher in 2017) (Table 1 and Table 2). Ethyl isobutyrate has been previously identified as important volatile in bourbon [16]. Ethyl butyrate (fruity/apples) also had a high average MF of 57.8% and was impacted by season and environment (higher in 2017 and Bunclody) (Table 1 and Table 2). Ethyl butyrate is commonly found in a wide range of spirits and is produced from ethanol and butyric acid during fermentation. Another ester ethyl-3-methylbutyrate (pineapple/apples) (also known as ethyl isovalerate) had an average MF of 54% and was impacted by season (higher in 2017). It is also an important odour compound in bourbon [16]. Two other esters were also aroma active but had MF values < 50% and therefore are less important. These again are products primarily of fermentation and are derived from their corresponding alcohols and acids. Ethyl decanoate (grape/apple/waxy) had an average MF of 39.5% and propyl acetate (pear/fruity) had an average MF of 20.3%. Both were not impacted by season, variety, or environment (Table 1 and Table 2). Ethyl decanoate has been previously found in whisk(e)y [7,48] and propyl acetate has been previously found in calvados [6] and cognac [17].

The acetals, 1,1-diethoxy-2-methyl-propane* (tentative identification) and 1,1-diethoxyethane, conferred fruity notes. The average MF values for 1,1-diethoxy-2-methyl-propane* and 1,1-diethoxyethane were 72.1 and 59.4%, respectively, and they were not influenced by season, environment, or variety. Acetals are characterised with more pleasant aromas (fruity/apple/pineapple/cherry) compared to their aldehyde precursors (pungent), and they are formed during distillation and are known to be significantly impacted by distillation conditions [17,49,50]. Another unknown compound (unknown compound 3) had a low average MF% of 24.1% and therefore is of less importance. It was characterised by a fruity/butter aroma and was impacted by variety (higher in Olympus) (Table 1 and Table 2).

#### 3.1.6. Grassy and Vegetal Odours

Three unknown compounds had high MF values within this category (Table 1). Unknown compound 10 (herbal/grass) had a very high contribution to the aroma of new make spirits based on its average MF value of 93.5%. Unknown compound 6 had an average MF value of 61.8% and was characterised by a cut grass/green-bell pepper aroma. The MF% values of the unknown compounds 6 and 10 were not impacted by season, variety, or environment. Unknown compound 9 with a grass aroma had a lower average MF value of 58.9% and was impacted by environment (higher at Athy) (Table 1 and Table 2).

Five aldehydes methional, heptanal, (E) 2-octenal, hexanal, and decanal were included in this category based on their aromas (Table 1). Methional belongs to the category of vegetal odours and is derived from methionine through Strecker degradation reactions. It was described as having a boiled potato odour and had a great significance on the overall aromatic profile with an average MF of 80.9%. Methional aroma intensity was impacted by season and environment (highest in 2017 and at Athy) (Table 1 and Table 2). In a previous study, methional was reported to have a negative impact on Spanish wine aroma by masking their fruity character [51]. However, as it has a very low odour threshold [24,42], it is an important compound even though potentially negatively impacting on aroma. Methional is also likely a precursor for dimethyl disulfide and dimethyl trisulfide in whisk(e)y [52], which are both problematic sulphur compounds that can carry over from distillation into the new make spirit. Heptanal had a seaweed/grass/rubber aroma and has previously been detected at trace levels in barley and malt for beer production [4]. The average MF values was 69.5% and the intensity was impacted by season and variety (highest in 2017 and Olympus) (Table 1 and Table 2). It is not a common compound in distilled spirits. The remaining aldehydes within this category had MF values <50% and therefore were less likely to be impactful aroma compounds. (E)-2-Octenal was characterised as having a vegetable/cabbage aroma but had a low average MF of 37%. (E)-2-octenal has been detected in barley [35] and along with (E)-2-nonenal and 1-decanal was associated with the sawdust character of wines aged in new make barrels [31]. The intensity was not impacted by season, variety, or environment. Hexanal had a characteristic green/vegetative odour and an average MF of 32.2%, and its intensity was impacted by variety (higher in Olympus). Hexanal is considered a key odorant in barley and malt and can be impacted by the malting process [4], fermentation conditions [53], and the distillation process [54]. The average MF value of decanal (grass/lemon) was 30.9%, and its value was not impacted by season, variety, or environment. It has been detected at trace levels in barley and malt for beer production [4].

The remaining volatile in this category is citronellyl acetate* (fresh/green) (only tentatively identified), which is an ester of a monoterpene citronellol likely derived directly from the barley. It is possible that the ester is formed during fermentation when the citronellol comes in contact with acetic acid. Its average MF value was 44.8%, but it was not impacted by season, variety, or environment. It is not a common compound in distilled spirits.

#### 3.1.7. Roasty Odours

Three volatile compounds were grouped as roasty (Table 1). Furfural is a Maillard reaction product and is formed during distillation [8,55] malting, kilning [56], and over the maturation process from wood [55,57]. It is mainly a feint (the last fraction from a distillation) component and has been detected in spirits produced from a range of raw materials [7,8,13,19,37,54,58,59,60], and has also been detected in wort and beer [61,62]. The concentration of furfural can be affected by the yeast strain [53] and the distillation method [8,54,55]. It is known to contribute to the aroma of distillates at low concentrations [55] and was described as having a marzipan, sweet, oily and grainy, or sweet and caramel aroma in wort and beer [53]. In this study, furfural was determined as the main compound that contributed to the roasty aromas of the new make spirit with an odour of baked/toasted almond and represents one of the most important odorants with an average MF of 85.5% (Table 1). Furfural was not impacted by season, variety, or environment.

Two unknown compounds (unknown compound 7 and unknown compound 2) had average MF values > 50% and therefore are important aroma active compounds in these new make spirit samples. Unknown compound 7 (coffee) had an average MF of 62.4% and was not influenced by season, variety, and environment, while unknown compound 2 (coffee) had an average MF of 55.7% and was also not influenced by environment, variety, or season (Table 1).

#### 3.1.8. Key Aroma Active Compounds

The eight most odorous aromatic volatiles of the new make spirit (Table 1) in order of importance based on MF values are as follows (all MF > 80%): (E)-2-nonenal (fried/toasted/fatty), unknown 10 (herbal/grass), 3-methyl-1-butanol (fermented/yeast/rancid), furfural (baked/toasted almond), ethyl hexanoate (pear/fruity), unknown 8 (lemon/cleaning fluid), β-damascenone (honey/plum), and methional (boiled potato). This is a diverse range of chemical classes incorporating aldehydes (including a furan and sulphur aldehyde), a ketone, an alcohol, an ester, and two unidentified compounds. Another fifteen aromatic volatiles with MF values (between 50 and 80%) are also likely impacting on new make spirit flavour but to a lesser extent, and they are as follows in order of importance: 2-phenylethanol (rose/floral), 1-octen-3-one (metallic/mineral/mushroom), 1,1-diethoxy-2-methyl-propane* (pineapple/cherry), 3-methylbutyl acetate (banana/fruity/melon), heptanal (seaweed/grass/rubber), ethyl isobutyrate (fruity/sweet/cherry), unknown 7 (coffee), unknown 5 (soil/herbal), unknown 6 (cut-grass/green-bell pepper), 3-methyl butanal (butter/cheese/chocolate), 1,1-diethyoxyethane (apple/fruity), unknown 9 (grass), 2-phenylethyl acetate (floral), ethyl butyrate (fruity/apple), 2-pentyl furan (gas/bad smell), unknown 2 (coffee), ethyl 3-methylbutyrate (pineapple/apple), and unknown 1 (soil). The most prominent chemical class were esters, with aldehydes, acetals, a furan, an alcohol, and a ketone also contributing; however, six of these compounds could not be identified.

**Figure 1:**
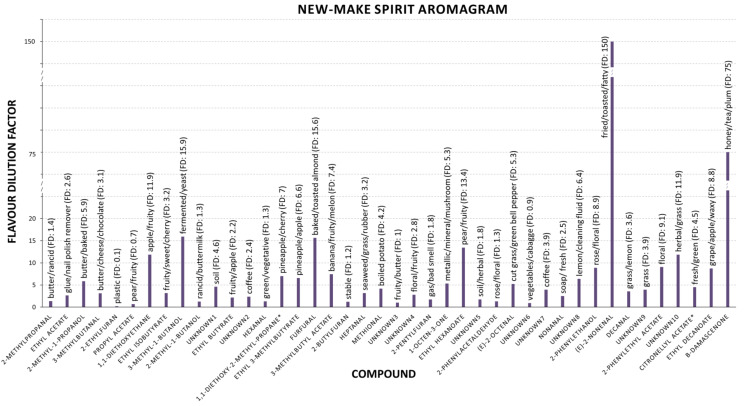
Aromagram of new make spirits illustrating the compounds, the mean value of flavour dilution factors (FD), and the odours of the compounds obtained by GCO analysis.

In addition, an aromagram was created with mean FD factors of the aroma active compounds to more easily visualise their respective individual contributions (Figure 1 and Supplementary Figure S2). The highest FD factors of the odorants were determined for (E)-2-nonenal (FD = 150) and β-damascenone (FD = 75) followed by compounds with much lower FD factors (FD < 20), such as 3-methyl-1-butanol (FD = 15.9), furfural (FD = 15.6), ethyl hexanoate (FD = 13.4), unknown 10 (FD = 11.8), 1,1-diethoxyethane (FD = 11.8), 2-phenylethyl acetate (FD = 9.8), and 2-phenylethanol (FD = 8.9).

It is noteworthy that both MF (Table 1) and FD approaches identified that (E)-2-nonenal, β-damascenone, 3-methyl-1-butanol, furfural, ethyl hexanoate, and unknown compound 10 as the six most impactful aroma compounds in all of these new make spirit samples. Of these six volatiles, their aroma perception was not influenced by variety or season, but (E)-2-nonenal and β-damascenone were impacted by environment (Table 2).

### 3.2. Sensory Evaluation of New Make Spirits

Significant differences in the intensity of sensory attributes were identified between season, variety, environment, and the interactions thereof (Table 3). For both years, variety had a significant effect on pungent and fresh fruit attributes, and environment had a significant effect on pungent, feinty/earthy, malty/biscuity, floral, fresh fruit, dried fruit, solventy, and oily finish attributes, while the interaction of variety x environment significantly affected pungent, feinty/earthy, floral, and fresh fruit attributes. Some sensory attributes were only significant for one season (Table 3).

In terms of variety, Olympus scored significantly higher (*p* < 0.001 in 2017 and *p* < 0.05 in 2018) for the sensory attributes pungent and fresh fruit (<0.01) across both seasons and for the malty/biscuity (*p* < 0.01) sensory attribute in 2018 in comparison to the Laureate variety. The Laureate variety scored significantly higher (*p* < 0.01) for the floral sensory attribute in 2018 only. Flavour differences between varieties has been previously reported in beer [20,63]. In this study, significant differences between the varieties were evident for only two sensory attributes (pungent and fresh fruit) in 2017 and for four (pungent, fresh fruit, malty/biscuit, and floral) in 2018 (Table 3). The lack of significance across a higher number of sensory attributes between both Laureate and Olympus varieties suggests that in this study, the impact of variety is of less significance in comparison to environment. This may be a direct result of genetic distance between the two chosen varieties, as Windes et al. suggested that genetic distance between barley varieties may be a significant variable in the differential flavour profiling of lager beer and that the variation was greater between genetic linkage groups than within genetic linkage groups [63]. As Laureate and Olympus varieties were developed within similar genetic backgrounds, both sharing a common ancestor (Quench SB) and originating from European germplasm, these may be less likely to produce different flavour attributes than varieties derived from a wider germplasm [64].

In terms of environment for both seasons, Athy scored significantly higher for the sensory attributes: pungent (*p* < 0.05 for 2017 and *p* < 0.001 for 2018), feinty/earthy (*p* < 0.001), malty/biscuity (*p* < 0.01 for 2017 and *p* < 0.001 for 2018), and oily finish (*p* < 0.05 for 2017 and *p* < 0.001 for 2018), and for cereal/grainy (*p* < 0.01), and for sulphury (*p* < 0.001) in 2018 (Table 3). Bunclody was significantly more associated with the sensory attributes floral (*p* < 0.001), fresh fruit (*p* < 0.01), and dried fruit (*p* < 0.01) attributes for both seasons, and for the green/grassy attribute in 2017 (Table 3). Both sites were selected based on their unique environmental attributes, with soil type being the fundamental difference between the two. In Athy, the predominant soil association was Elton, a limestone-based soil, which is intrinsically alkaline as shown by the increased pH levels through the subsoil structure, whereas the soil at the Bunclody is acidic-neutral (Supplementary Table S1). Soil pH is a key factor in nutrient uptake and affects availability by changing the form of the macro- and micronutrients in the soil [60], which subsequently impacts on barley growth.

Variety x environment interactions were significant for five sensory attributes in 2017 and for seven in 2018 (Table 3). For both seasons, the sensory attribute pungent aroma was significantly higher (*p* < 0.05 for 2017 and *p* < 0.01 for 2018) in the Olympus variety at both environments in comparison to that produced from the Laureate variety at both environments. In 2017, the feinty/earthy and cereal/grainy sensory attributes were significantly higher (*p* < 0.01) in the Olympus variety from Athy. In 2017, the floral sensory attribute was significantly higher (*p* < 0.05) in the Laureate variety from Bunclody, whereas also in 2017, the sensory attribute fresh fruit was significantly higher (*p* < 0.05) in the Olympus variety from Bunclody. In 2018, the sensory attributes oily finish and sulphury aromas were significantly higher (*p* < 0.05) from the Olympus and Laureate varieties from Athy compared to both Laureate and Olympus varieties from Bunclody. In addition, in 2018, the solventy sensory attribute was significantly lower in the Olympus variety from Athy (*p* < 0.01) compared to all samples. Among the significant interactions, the new make spirit from the Olympus variety from Athy was characterised as more pungent, feinty/earthy, cereal-grainy, oily-finish, and sulphury compared to all other variety x environment interactions. Likewise, the interaction of the new make spirit from the Laureate variety from Bunclody was described as more floral and solventy. Therefore, the significance of the variety x environment interaction suggests that both barley variety and environment contribute to the flavour of new make spirit, and that specific variety and environment combinations may further impact flavour profiles.

### 3.3. Chemometric Analysis of Sensory and Aroma Data

The MF values from the GCO data for a number of aroma active compounds correlated with the sensory attributes (Table 4).

The impact of variety on the aromatic sensory properties of new make spirit was greater for Olympus than Laureate as evident by the significantly higher (*p* < 0.05) perception for heptanal, hexanal, unknown compound 3, and 2-phenylacetaldehyde (Table 2). However, neither heptanal, hexanal, or 2-phenylacetaldehyde were correlated with any sensory attributes, but the unknown compound 3 was positively correlated with cereal/grainy (*p* < 0.05) (Table 4). No aromatic volatile compounds were significantly higher in the Laureate variety (Table 2). Overall, these results highlight that variety did not contribute much difference to the aromatic sensory properties of the new make spirit, as only one volatile correlated with one sensory attribute. These results highlight a similar trend found in the descriptive sensory data.

The influence of environment on the aromatic sensory profile of the new make spirit was much more pronounced than variety. Samples from Athy were characterised with a higher perception (*p* < 0.05) of (E)-2-nonenal (Table 2), which was positively correlated (*p* < 0.05) with sweet (Table 4). These Athy samples also had a higher perception (*p* < 0.05) of methional (Table 2), which was positively correlated (*p* < 0.01) with cereal/grainy and negatively correlated (*p* < 0.05) with green/grassy, fresh/fruit, and soapy (Table 4). The samples from Athy also had a higher perception (*p* < 0.05) for unknown compound 9 (Table 2), which was negatively (*p* < 0.05) associated with floral, fresh fruit, and dried fruit (Table 4). The Athy samples also had higher perceptions (*p* < 0.05) for both 2-butylfuran and 2-pentylfuran (Table 2). 2-Butylfuran was positively correlated with the feinty/earthy and oily finish (*p* < 0.01), soapy, sweet, sour, and stale/mouldy (*p* < 0.05) and negatively with solventy (*p* < 0.05) attributes (Table 4). 2-Pentyfuran was positively correlated with cereal/grainy (*p* < 0.05) and negatively with fresh fruit (*p* < 0.01) (Table 4). The new make spirit from Bunclody was characterised by higher perceptions (*p* < 0.05) of β-damascenone and unknown compound 5 (Table 2). β-Damascenone was positively correlated with dried fruit (*p* < 0.01) and solventy (*p* < 0.05), and negatively with malty/biscuit (*p* < 0.01), feinty/earthy, sour, sulphury (*p* < 0.05) attributes (Table 4). Unknown compound 5 is positively associated with solventy (*p* < 0.05) and negatively with oily finish, sour, sulphury (*p* < 0.001), feinty/earthy (*p* < 0.01), and pungent, malty/biscuits, soapy, sweet (*p* < 0.05) attributes (Table 4). Thus, overall, the new make spirit from Athy is more positively associated with sweet, cereal/grainy, feinty/earthy, oily finish, soapy, sour, stale, and mouldy sensory attributes and the new make spirit from Bunclody was more associated with dried fruit and solventy attributes. These results also correspond to the descriptive sensory data.

The impact of season on the aromatic sensory profiles of the new make spirit highlighted differences between 2017 and 2018. In 2017, methional, unknown compound 5, unknown compound 1, ethyl isobutyrate, ethyl-3-methylbutyrate, and unknown compound 4 were perceived (*p* < 0.05) as more intense than in 2018. Methional was positively correlated with cereal/grainy (*p* < 0.01) and negatively correlated with green/grassy, fresh fruit, and soapy (*p* < 0.05) attributes. Unknown 5 was positively correlated with solventy (*p* < 0.05) and negatively correlated with sulphury, sour, oily/fishy (*p* < 0.001), feinty/earthy (*p* < 0.01), and pungent, malty/biscuity, soapy, sweet (*p* < 0.05) attributes. Unknown 1 was negatively correlated with soapy (*p* < 0.05), and ethyl isobutyrate was negatively correlated with feinty/earthy, soapy, oily finish, and sour (*p* < 0.05) attributes. Ethyl 3-methylbutyrate was positively correlated with cereal/grainy (*p* > 0.05) and negatively correlated with soapy (*p* < 0.01) attributes. Unknown 4 was positively correlated with cereal/grainy (*p* < 0.05), and negatively correlated with soapy (*p* < 0.01), oily finish, and sour (*p* < 0.05) attributes. In 2018, the following compounds were perceived at greater intensity (*p* < 0.05) than in 2017; unknown 8, 1-octen-3-one, 2-butylfuran, and 2-phenylacetaldehyde. Unknown 8 was positively associated with sour (*p* < 0.01), feinty/earthy, soapy, oily finish (*p* < 0.05), and 1-octen-3-one was positively associated with sour, (*p* < 0.001), sulphury, feinty/earthy, oily finish (*p* < 0.01), and malty/biscuity, sweet (*p* < 0.05), and negatively correlated with solventy (*p* < 0.01) attributes. 2-Butylfuran was positively associated with feinty/earthy, oily inish (*p* < 0.01), and soapy, sweet, sour, stale/mouldy (*p* < 0.05) attributes and negatively correlated with solventy (*p* < 0.05) attributes. 2-Phenylacetaldehyde was not associated with any sensory attribute. These results highlight that the new make spirit produced in 2018 had more aromatic sensory associations than that produced in 2017. The strongest associations for new make spirit in 2017 were cereal/grainy and solventy, while those from 2018 were feinty/earthy, oily finish, sour, soapy, and sweet and lesser so with sulphury, malty/biscuity, and stale/mouldy. These results highlight that season has an impact on the aromatic sensory perception of new makes spirits that is greater than the varieties in this study.

## 4. Conclusions

This study attempted to determine the influence of terroir on the flavour of new make spirits by assessing the contribution of barley variety and its growth environment over two seasons through sensory and olfactometry analysis. It was noted that variety, environment, and the interaction of variety x environment impacted the sensory character of the new make spirits, with pungent and fresh fruit sensory attributes impacted by all factors (variety, environment, and season). However, the impact of environment and the interaction of variety x environment were more pronounced than variety alone on the sensory attributes across both seasons. Forty-two volatile compounds were detected as potential odorants contributing to the flavour of new make spirits; however, eight were deemed to be the most influential: (E)-2-nonenal, β-damascenone, 3-methyl-1-butanol, furfural, ethyl hexanoate, and one unidentified compound (unknown 10 with a herbal/grass character). Another fifteen also impacted on aroma but to a lesser extent, and these consisted of mainly esters, although six compounds could not be identified due to co-elution, low abundance, or by the fact that they were below limits of detection by mass spectrometry. Chemometric analysis of the volatile and sensory data also concluded that both environment and season had a greater impact on the aromatic sensory character of the new make spirits than variety alone. The environments were chosen in this study based on different soil and climate conditions, where the barley varieties were chosen based on common commercial varieties in use in Ireland at the time of the study, and these varieties share similar genetic heritage that may have limited an impact on flavour diversity in the new make spirit.

This study has clearly demonstrated variations in the contribution of the aroma active volatiles and sensory attributes in these new make spirits, which reflect changes in barley growth in relation to environmental elements including soil nutrients and prevailing seasonal weather patterns; therefore, it reveals a “terroir” effect. This has not been previously determined and creates the possibility of producing whisk(e)y from different “vintage” with new make spirit that encompasses the factors impacting on the growth of the barley variety as well as the subsequent processing parameters. Further research is required to better understand the specific environmental impact on barley growth and the management and processing thereof with respect to the genetic, physiological, and metabolic mechanisms contributing to the terroir expression of new make spirit but also in whisk(e)y to determine the significance of terroir post the maturation process.

## Figures and Tables

**Table 1:** Aroma active compounds and their modified frequency percentage (MF%) average values detected in new make spirit by gas chromatography oflactometry (GCO)/MS analysis.

Compound	Cas No	RT ^1^	LRI	Chemical Class	Descriptor	Odour Type	MF% Average
Nonanal	124-19-6	18.07	1147.4	Aldehyde	soap/fresh	chemical	42.4
Ethyl acetate	141-78-6	4.30	642.2	Ester	glue/nail polish remover	chemical	12.0
2-Ethylfuran	3208-16-0	5.94	717.1	Furan	plastic	chemical	12.0
1-Octen-3-one	4312-99-6	14.70	1018.5	Ketone	metallic/mineral/mushroom	earthy	72.6
Unknown5	-	16.27	1073.0	-	soil/herbal	earthy	61.9
2-Pentylfuran	3777-69-3	14.36	1008.7	Furan	gas/bad smell	earthy	56.6
Unknown1	-	8.25	796.5	-	soil	earthy	52.1
2-Butylfuran	4466-24-4	12.20	932.7	Furan	stable	earthy	30.4
(E)-2-Nonenal	18829-56-6	19.80	1219.8	Aldehyde	fried/toasted/fatty	fatty	94.2
3-Methyl-1-butanol (Isoamyl alcohol)	123-51-3	8.08	789.1	Alcohol	fermented/yeast	fatty	87.1
3-Methylbutanal (Isovaleraldehyde)	590-86-3	5.33	693.4	Aldehyde	butter/cheese/chocolate	fatty	60.0
2-Methyl-1-butanol	137-32-6	8.15	792.5	Alcohol	rancid/buttermilk	fatty	47.9
2-Methyl-1-propanol (Isobutyl alcohol)	78-83-1	5.15	685.9	Alcohol	butter/baked	fatty	40.9
2-Methylpropanal (Isobutyraldehyde)	78-84-2	3.20	598.6	Aldehyde	butter/rancid	fatty	16.8
β-Damascenone	23726-93-4	23.03	1452.7	Ketone	honey/tea/plum	floral	82.3
2-Phenylethanol	60-12-8	19.30	1194.5	Alcohol	rose/floral	floral	77.9
2-Phenylethyl acetate	103-45-7	21.30	1311.3	Ester	floral	floral	58.8
Unknown4	-	13.96	991.7	-	floral/fruity	floral	48.8
2-Phenylacetaldehyde	122-78-1	17.16	1108.2	Aldehyde	rose/floral	floral	26.6
Ethyl hexanoate	123-66-0	14.89	1025.3	Ester	pear/fruity	fruity	84.9
Unknown8	-	19.22	1191.5	-	lemon/cleaning fluid	fruity	84.2
1,1-Diethoxy-2-methyl-propane *	1741-41-9	10.55	874.3	Acetal	pineapple/cherry	fruity	72.1
3-Methylbutyl acetate (Isoamyl acetate)	123-92-2	11.50	905.5	Ester	banana/fruity/melon	fruity	70.4
Ethyl isobutyrate	97-62-1	7.90	784.9	Ester	fruity/sweet/cherry	fruity	62.7
1,1-Diethoxyethane	105-57-7	6.81	746.6	Acetal	apple/fruity	fruity	59.4
Ethyl butyrate	105-54-4	9.16	827.7	Ester	fruity/apple	fruity	57.8
Ethyl 3-methylbutyrate (Ethyl Isovalerate)	108-64-5	10.70	879.6	Ester	pineapple/apple	fruity	54.0
Ethyl decanoate	110-38-3	22.73	1423.1	Ester	grape/apple/waxy	fruity	39.6
Unknown3	-	13.80	985.7	-	fruity/butter	fruity	24.1
Propyl acetate	109-60-4	6.71	744.4	Ester	pear/fruity	fruity	20.3
Unknown10	-	21.41	1318.0	-	herbal/grass	grassy	93.5
Heptanal	111-71-7	12.50	941.5	Aldehyde	seaweed/grass/rubber	grassy	69.5
Unknown6	-	17.28	1114.5	-	cut grass/green bell pepper	grassy	61.9
Unknown9	-	20.89	1278.5	-	grass	grassy	58.9
Citronellyl acetate *	150-84-5	22.26	1384.3	Ester	fresh/green	grassy	44.8
Decanal	112-31-2	20.20	1251.6	Aldehyde	grass/lemon	grassy	30.9
Methional	3268-49-3	13.31	968.8	Aldehyde	boiled potato	vegetal	80.9
(E)-2-Octenal	2548-87-0	17.35	1118.5	Aldehyde	vegetable/cabagge	vegetal	37.0
Hexanal	66-25-1	9.50	838.7	Aldehyde	green/vegetative	vegetal	32.2
Furfural	98-01-1	11.20	896.7	Furan	baked/toasted almond	roasty	85.5
Unknown7	-	17.73	1132.5	-	coffee	roasty	62.5
Unknown2	-	9.22	828.5	-	coffee	roasty	55.7

* Tentative identification or could be an isomer of this compound, ^1^ RT: Retention Time, LRI: Linear Retention Index, MF%: % Modified Frequency.

**Table 2:** Aroma active compounds affected by variety (Laureate and Olympus), environment (Athy and Bunclody), or season (2017 and 2018).

Compound	Attribute	Odour Type	Variety MF%		Environment MF%		Season MF%	
			Laureate	Olympus	Athy	Bunclody	2017	2018
1-Octen-3-one	metallic/mineral/mushroom	earthy	72.4 ^a^	72.7 ^a^	75.8 ^a^	69.3 ^a^	63.6 ^b^	81.5 ^a^
Unknown 5	soil/herbal	earthy	64.5 ^a^	59.3 ^a^	52.7 ^b^	71.1 ^a^	83.1 ^a^	40.6 ^b^
2-Pentylfuran	gas/bad smell	earthy	62.7 ^a^	50.6 ^a^	66.3 ^a^	47.0 ^b^	69.5 ^a^	43.8 ^b^
Unknown 1	soil	earthy	63.7 ^a^	40.6 ^a^	48.8 ^a^	55.5 ^a^	71.0 ^a^	33.3 ^b^
2-Butylfuran	stable	earthy	29.8 ^a^	31.0 ^a^	38.0 ^a^	22.8 ^b^	14.4 ^b^	46.4 ^a^
(E)-2-Nonenal	fried/toasted/fatty	fatty	94.3 ^a^	94.1 ^a^	97.1 ^a^	91.2 ^b^	94.2 ^a^	94.2 ^a^
β-Damascenone	honey/tea/plum	floral	85.8 ^a^	78.7 ^a^	73.7 ^b^	90.8 ^a^	89.3 ^a^	75.3 ^a^
Unknown 4	floral/fruity	floral	43.3 ^a^	54.3 ^a^	47.3 ^a^	50.3 ^a^	60.0 ^a^	37.7 ^b^
2-Phenylacetaldehyde	rose/floral	floral	16.4 ^b^	36.8 ^a^	23.5 ^a^	29.7 ^a^	11.8 ^b^	41.4 ^a^
Unknown 8	lemon/cleaning fluid	fruity	84.1 _a_	84.3 ^a^	89.5 ^a^	79.0 ^a^	75.8 ^b^	92.7 ^a^
Ethyl isobutyrate	fruity/sweet/cherry	fruity	64.3 _a_	61.1 ^a^	59.1 ^a^	66.3 ^a^	70.1 ^a^	55.3 ^b^
Ethyl 3-methylbutyrate (Ethyl Isovalerate)	pineapple/apple	fruity	49.3 _a_	58.7 ^a^	59.4 ^a^	48.6 ^a^	70.1 ^a^	37.9 ^b^
Unknown 3	fruity/butter	fruity	9.6 ^b^	38.5 ^a^	24.1 ^a^	24.0 ^a^	33.7 a	14.4 ^a^
Heptanal	seaweed/grass/rubber	grassy	57.7 ^b^	81.3 ^a^	72.9 ^a^	66.0 ^a^	78.5 a	60.5 ^a^
Unknown 9	grass	grassy	61.1 ^a^	56.7 ^a^	69.1 ^a^	48.6 ^b^	60.7 a	57.1 ^a^
Methional	boiled potato	vegetal	80.9 ^a^	80.9 ^a^	89.3 ^a^	72.5 ^b^	92.7 ^a^	69.1 ^b^
Hexanal	green/vegetative	vegetal	9.6 ^b^	54.7 ^a^	33.3 ^a^	31.1 ^a^	26.2 a	38.1 ^a^

^a, b^: significant differences in MF% among varieties, environments or seasons are indicated by different letters (Tukey’s test, *p* < 0.05).

**Table 3:** Mean separation analysis and significance level of main effects and key interaction terms in the analyses of variance (ANOVAs) on sensory descriptors from new make spirit samples from two varieties (Laureate and Olympus) grown in two environments (Athy and Bunclody) during 2017 and 2018 seasons.

	Intensity										
	Variety			Environment			Variety × Environment				
Sensory Attribute	Laureate	Olympus	Significant Level	Athy	Bunclody	Significant Level	Laureate × Athy	Laureate × Bunclody	Olympus × Athy	Olympus × Bunclody	Significant Level
	**Season 2017**										
Pungent	1.85 ^b^	2.50 ^a^	***	2.25 ^a^	2.10 ^b^	*	1.85 ^b,c^	1.56 ^c^	2.66 ^a^	2.33 ^a,b^	*
Feinty/Earthy	0.74 ^a^	0.99 ^a^	---	1.16 ^a^	0.57 ^b^	***	0.87 ^b^	0.61 ^b^	1.45 ^a^	0.53 ^b^	**
Cereal/Grainy	2.30 ^a^	2.46 ^a^	---	2.42 ^a^	2.34 ^a^	---	2.15 ^a^	2.46 ^a,b^	2.70 ^a^	2.22 ^b^	**
Malty/Biscuity	2.47 ^a^	2.44 ^a^	---	2.70 ^a^	2.21 ^b^	**	2.63 ^a,b^	2.31 ^b^	2.76 ^a^	2.12 ^b^	---
Green/Grassy	0.90 ^a^	0.80 ^a^	---	0.49 ^b^	1.21 ^a^	***	0.47 ^b^	1.33 ^a^	0.51 ^b^	1.08 ^a^	---
Floral	1.38 ^a^	1.07 ^a^	---	0.79 ^b^	1.66 ^a^	***	0.87 ^c^	1.90 ^a^	0.71 ^c^	1.43 ^b^	*
Fresh Fruit	2.41 ^b^	2.98 ^a^	**	2.55 ^b^	2.83 ^a^	**	2.35 ^b^	2.47 ^b^	2.76 ^b^	3.20 ^a^	*
Dried Fruit	2.40 ^a^	2.60 ^a^	---	2.25 ^b^	2.74 ^a^	**	2.12 ^c^	2.67 ^a,b^	2.38 ^b,c^	2.81 ^a^	---
Soapy	1.19 ^a^	1.22 ^a^	---	1.29 ^a^	1.13 ^a^	---	1.36 ^a^	1.02 ^a^	1.21 ^a^	1.23 ^a^	---
Solventy	2.00 ^a^	2.20 ^a^	---	1.91 ^b^	2.28 ^a^	*	1.85 ^a^	2.15 ^a^	1.98 ^a^	2.42 ^a^	---
Sweet	2.15 ^a^	2.08 ^a^	---	2.19 ^a^	2.05 ^a^	---	2.17 ^a^	2.13 ^a^	2.21 ^a^	1.96 ^a^	---
Oily finish	0.30 ^a^	0.45 ^a^	---	0.53 ^a^	0.21 ^b^	*	0.42 ^a,b^	0.17 ^b^	0.65 ^a^	0.25 ^b^	---
Sour	0.13 ^a^	0.04 ^a^	---	0.15 ^a^	0.02 ^a^	---	0.25 ^a^	0.01 ^a^	0.05 ^a^	0.03 ^a^	---
Sulphury	0.16 ^a^	0.15 ^a^	---	0.17 ^a^	0.15 ^a^	---	0.10 ^a^	0.23 ^a^	0.25 ^a^	0.06 ^a^	---
Stale/Mouldy	---	---	---	---	---	---	---	---	---	---	---
	**Season 2018**										
Pungent	2.49 ^b^	2.77 ^a^	*	3.12 ^a^	2.14 ^b^	***	2.77 ^b^	2.21 ^c^	3.46 ^a^	2.07 ^c^	**
Feinty/Earthy	1.77 ^a^	1.94 ^a^	---	2.22 ^a^	1.48 ^b^	***	1.99 ^b^	1.54 ^c^	2.45 ^a^	1.43 ^c^	*
Cereal/Grainy	1.56 ^a^	1.68 ^a^	---	1.77 ^a^	1.47 ^b^	**	1.66 ^a,b^	1.45 ^b^	1.88 ^a^	1.48 ^b^	---
Malty/Biscuity	2.64 ^b^	2.92 ^a^	**	3.13 a	2.43 ^b^	***	3.08 ^a^	2.19 ^c^	3.18 ^a^	2.66 ^b^	---
Green/Grassy	1.52 ^a^	1.30 ^a^	---	1.32 a	1.50 ^a^	---	1.47 ^a^	1.57 ^a^	1.18 ^a^	1.42 ^a^	---
Floral	1.92 ^a^	1.33 ^b^	**	1.02 b	2.24 ^a^	***	1.33 ^c^	2.52 ^a^	0.71 ^d^	1.96 ^b^	*
Fresh Fruit	2.87 ^b^	3.21 ^a^	**	2.62 b	3.46 ^a^	***	2.49 ^c^	3.26 ^b^	2.76 ^c^	3.66 ^a^	*
Dried Fruit	2.49 ^a^	2.22 ^a^	---	2.15 b	2.56 ^a^	**	2.38 ^a^	2.60 ^a^	1.92 ^b^	2.51 ^a^	---
Soapy	1.87 ^a^	1.65 ^a^	---	1.69 a	1.83 ^a^	---	1.89 ^a^	1.86 ^a^	1.49 ^a^	1.81 ^a^	---
Solventy	1.71 a	1.66 ^a^	---	1.57 b	1.81 ^a^	*	1.74 ^a^	1.69 ^a,b^	1.40 ^b^	1.93 ^a^	**
Sweet	2.30 ^a^	2.31 ^a^	---	2.40 a	2.22 ^a^	---	2.29 ^a,b^	2.31 ^a,b^	2.50 ^a^	2.13 ^b^	---
Oily finish	1.71 ^a^	1.76 ^a^	---	2.15 a	1.31 ^b^	***	1.97 ^a^	1.46 ^b^	1.97 ^a^	1.17 ^b^	*
Sour	0.64 ^a^	0.68 ^a^	---	0.77 a	0.56 ^a^	---	0.75 ^a^	0.54 ^a^	0.79 ^a^	0.58 ^a^	---
Sulphury	0.94 ^a^	1.02 ^a^	---	1.37 a	0.59 ^b^	***	1.33 ^a^	0.55 ^b^	1.42 ^a^	0.63 ^b^	*
Stale/Mouldy	0.36 ^a^	0.28 ^a^	---	0.30 a	0.34 ^a^	---	0.22 ^a^	0.50 ^a^	0.38 ^a^	0.18 ^a^	---

^a, b, c, d^: significant differences in sensory scores among varieties, environment, and interaction terms by season are indicated by different letters (Student’s *t*-test, *p* < 0.05 (*); *p* < 0.01 (**); *p* < 0.001 (***)).

**Table 4:** Pearson’s correlation coefficients obtained between sensory attributes and MF% values of aroma active compounds.

	Aroma Active Compounds	Sensory Attributes														
		Pungent	Feinty/Earthy	Cereal/Grainy	Malty/Biscuity	Green/Grassy	Floral	Fresh Fruit	Dried Fruit	Soapy	Solventy	Sweet	Oily Finish	Sour	Sulphury	Stale/Mouldy
1	3-Methylbutanal (Isovaleraldehyde) (butter/cheese/chocolate)	-	-	-	-	0.726 *	-	-	-	-	-	-	-	-	-	-
2	Propyl acetate (pear/fruity)	-	-	-	-	-	−0.759 *	-	-	-	-	-	-	-	-	-
3	1,1-Diethoxyethane (apple/fruity)	-	-	-	-	-	-	-	-	-	0.734 *	-	-	-	-	−0.775 *
4	Ethyl isobutyrate (fruity/sweet/cherry)	-	−0.798 *	-	-	-	-	-	-	−0.819 *	-	-	−0.795 *	-0.750 *	-	-
5	3-Methyl-1-butanol (Isoamyl alcohol) (fermented/yeast)	-	-	-	-	-	-	−0.719 *	-	-	-	-	-	-	-	-
6	Unknown 1 (soil)	-	-	-	-	-	-	-	-	−0.708 *	-	-	-	-	-	-
7	Ethyl butyrate (fruity/apple)	-	-	-	-	-	-	-	-	-	0.8138 *	−0.722 *	−0.753*	−0.761 *	-	-
8	Unknown 2 (coffee)	−0.714*	-	-	-	-	-	-	-	-	-	-	-	-0.708 *	-	-
9	Ethyl 3-methylbutyrate (Ethyl Isovalerate) (pineapple/apple)	-	-	0.823 *	-	-	-	-	-	−0.891 **	-	-	-	-	-	-
10	Furfural (baked/toasted almond)	-	-	-	0.779 *	-	-	-	-	-	-	-	-	0.726 *	0.724 *	-
11	2-Butylfuran (stable)	-	0.881 **	-	-	-	-	-	-	0.782 *	−0.823 *	0.770 *	0.870 **	0.796 *	-	0.077 *
12	Methional (boiled potato)	-	-	0.847 **	-	−0.730 *	-	−0.722 *	-	−0.767 *	-	-	-	-	-	-
13	Unknown 3 (fruity/butter)	-	-	0.721 *	-	-	-	-	-	-	-	-	-	-	-	-
14	Unknown 4 (floral/fruity)	-	-	0.724 *	-	-	-	-	-	−0.868 **	-	-	−0.733 *	−0.730 *	-	-
15	2-Pentylfuran (gas/bad smell)	-	-	0.801 *	-	-	-	−0.837 **	-	-	-	-	-	-	-	-
16	1-Octen-3-one (metallic/mineral/mushroom)	-	0.886 **	-	0.743 *	-	-	-	-	-	−0.865 **	0.8139 *	0.884**	0.915 ***	0.888 **	-
17	Unknown 5 (soil/herbal)	−0.715 *	-0.930 **	-	−0.782 *	-	-	-	-	−0.741 *	0.781 *	−0.746 *	−0.966 ***	−0.95 ***	−0.966 ***	-
18	Unknown 7 (coffee)	-	-	-	-	−0.716 *	-	-	-	-	-	-	-	-	-	-
19	Unknown 8 (lemon/cleaning fluid)	-	0.798 *	-	-	-	-	-	-	0.815 *	-	-	0.808 *	0.784 **	-	-
20	(E)-2-Nonenal (fried/toasted/fatty)	-	-	-	-	-	-	-	-	-	-	0.737 *	-	-	-	-
21	Unknown 9 (grass)	-	-	-	-	-	−0.736 *	−0.761 *	−0.810 *	-	-	-	-	-	-	-
22	β-Damascenone honey/tea/plum	-	−0.735 *	-	−0.857 **	-	-	-	0.866 **	-	0.727 *	-	-	−0.740 *	−0.709 *	-

Significant correlations are indicated by *p* < 0.05 (*); *p* < 0.01 (**); *p* < 0.001 (***).

## Data Availability

The data presented in this study are available in (www.mdpi.com/xxx Link to Supplementary Data).

## Supplementary Materials

The following are available online at https://www.mdpi.com/2304-8158/10/2/443/s1, Table S1: Average soil nutrients from Athy, Co. Kildare and Bunclody, Co. Wexford across 2017 and 2018 seasons., Table S2: Average weather measurements from Athy, Co. Kildare and Bunclody, Co. Wexford across 2017 and 2018 seasons, Table S3: Odour description of the pens from the Sniffin’ Sticks (Test Blue Kit), Table S4: Odours detected in new make spirits and used for the GCO analysis. Table S5: Standard mix solution in 20% of ethanol in water. Figure S1: Chromatogram of new make spirit sample analysed by GCO. Figure S2: Aromagrams of flavor dilution (FD) factors for new make spirits from (A1) Laureate variety/Athy in 2017, (A2) Laureate variety/Athy in 2018, (B1) Olympus variety/Athy in 2017, (B2) Olympus variety/Athy in 2018, (C1) Laureate variety/Bunclody in 2017, (C2) Laureate variety/Bunclody in 2018, (D1) Olympus variety/Bunclody in 2017, (D2) Olympus variety/Bunclody in 2018.
