# Supplementary material for: The Impact of Terroir on the Flavour of Single Malt Whisk(e)y New Make Spirit
Source: Foods. 2021 Feb 18;10(2):443. doi: 10.3390/foods10020443 (PMC7922972; doi:10.3390/foods10020443)
Supplement: Supplementary file 1 [file foods-10-00443-s001.pdf]

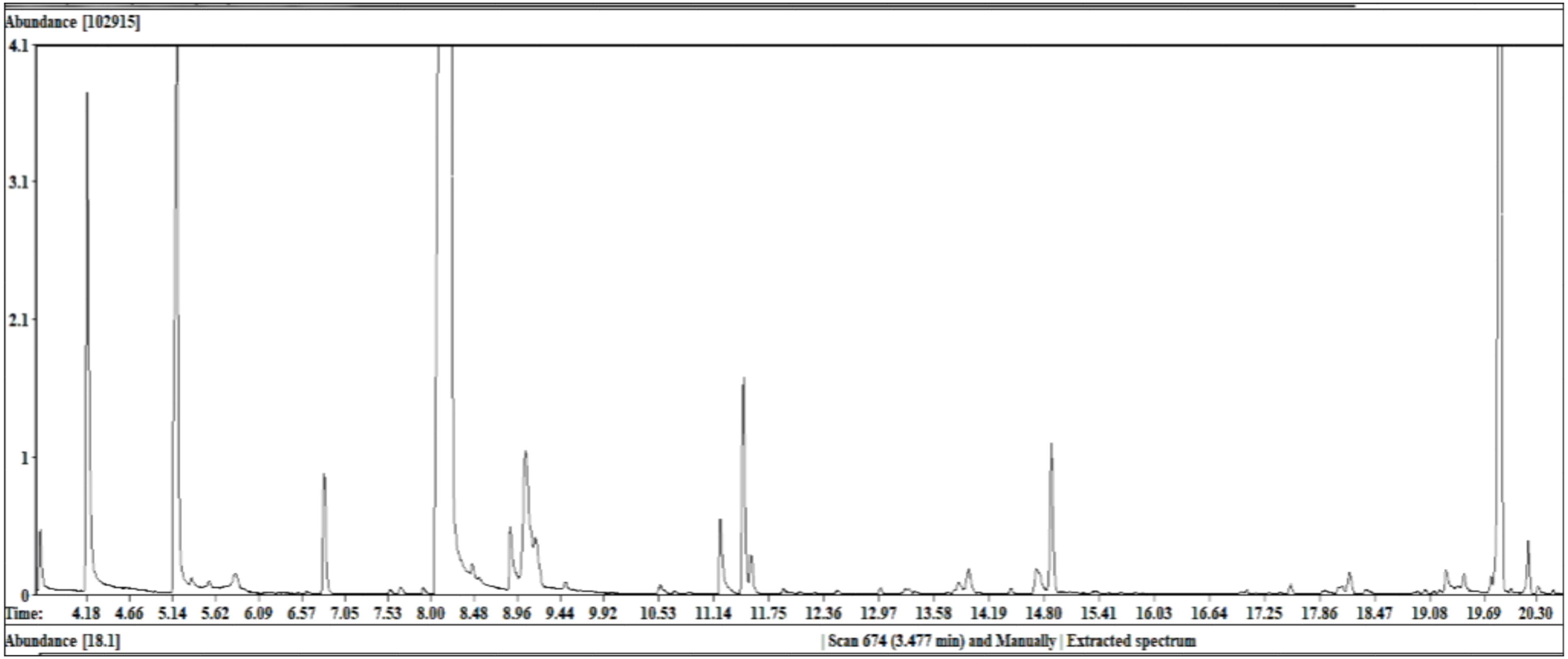

Supplementary Figure 1: Chromatogram of new make spirit sample analysed by GCO.

Supplementary Figure 2. Aromagrams of flavor dilution (FD) factors for new make spirits from (A1) Laureate variety/Athy in 2017, (A2) Laureate variety/Athy in 2018, (B1) Olympus variety/Athy in 2017, (B2) Olympus variety/Athy in 2018, (C1) Laureate variety/Bunclody in 2017, (C2) Laureate variety/Bunclody in 2018, (D1) Olympus variety/Bunclody in 2017, (D2) Olympus variety/Bunclody in 2018.

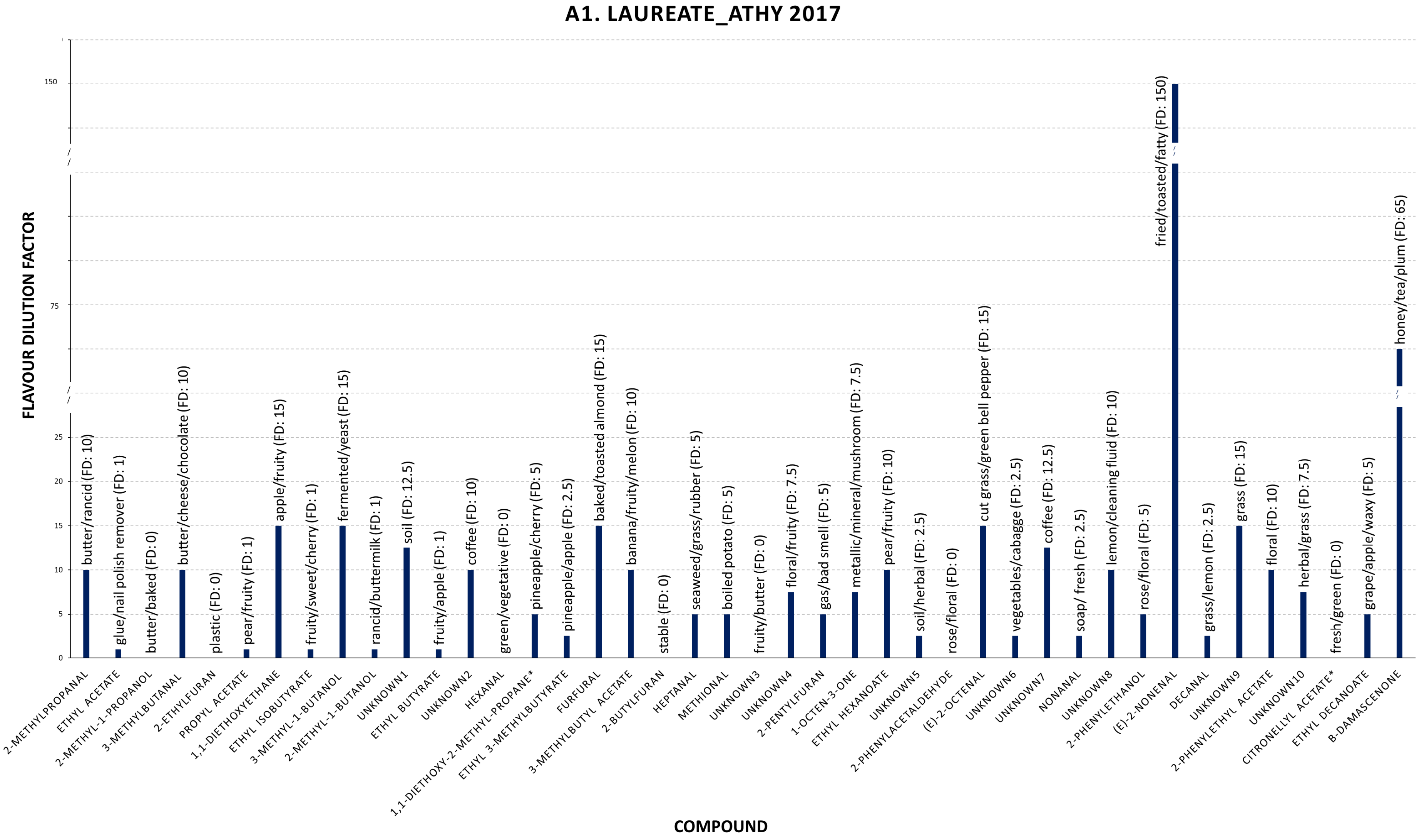
A1. LAUREATE_ATHY 2017
FLAVOUR DILUTION FACTOR
150
75
25
20
15
10
5
0
butter/rancid (FD: 10)
glue/nail polish remover (FD: 1)
butter/baked (FD: 0)
butter/cheese/chocolate (FD: 10)
plastic (FD: 0)
pear/fruity (FD: 1)
apple/fruity (FD: 15)
fruity/sweet/cherry (FD: 1)
fermented/yeast (FD: 15)
rancid/buttermilk (FD: 1)
soil (FD: 12.5)
fruity/apple (FD: 1)
coffee (FD: 10)
green/vegetative (FD: 0)
pineapple/cherry (FD: 5)
pineapple/apple (FD: 2.5)
baked/toasted almond (FD: 15)
banana/fruity/melon (FD: 10)
stable (FD: 0)
seaweed/grass/rubber (FD: 5)
boiled potato (FD: 5)
fruity/butter (FD: 0)
floral/fruity (FD: 7.5)
gas/bad smell (FD: 5)
metallic/mineral/mushroom (FD: 7.5)
pear/fruity (FD: 10)
soil/herbal (FD: 2.5)
rose/floral (FD: 0)
cut grass/green bell pepper (FD: 15)
vegetables/cabagge (FD: 2.5)
coffee (FD: 12.5)
soap/ fresh (FD: 2.5)
lemon/cleaning fluid (FD: 10)
rose/floral (FD: 5)
fried/toasted/fatty (FD: 150)
grass/lemon (FD: 2.5)
grass (FD: 15)
floral (FD: 10)
herbal/grass (FD: 7.5)
fresh/green (FD: 0)
grape/apple/waxy (FD: 5)
honey/tea/plum (FD: 65)
2-METHYLPROPANAL
ETHYL ACETATE
2-METHYL-1-PROPANOL
3-METHYLBUTANAL
2-ETHYLFURAN
PROPYL ACETATE
1,1-DIETHOXYETHANE
ETHYL ISOBUTYRATE
3-METHYL-1-BUTANOL
2-METHYL-1-BUTANOL
UNKNOWN1
ETHYL BUTYRATE
UNKNOWN2
HEXANAL
1,1-DIETHOXY-2-METHYL-PROPANE*
ETHYL 3-METHYLBUTYRATE
FURFURAL
3-METHYLBUTYL ACETATE
2-BUTYLFURAN
HEPTANAL
METHIONAL
UNKNOWN3
UNKNOWN4
2-PENTYLFURAN
1-OCTEN-3-ONE
ETHYL HEXANOATE
UNKNOWN5
2-PHENYLACETALDEHYDE
(E)-2-OCTENAL
UNKNOWN6
UNKNOWN7
NONANAL
UNKNOWN8
2-PHENYLETHANOL
(E)-2-NONENAL
DECANAL
UNKNOWN9
2-PHENYLETHYL ACETATE
UNKNOWN10
CITRONELLYL ACETATE*
ETHYL DECANOATE
B-DAMASCENONE
COMPOUND

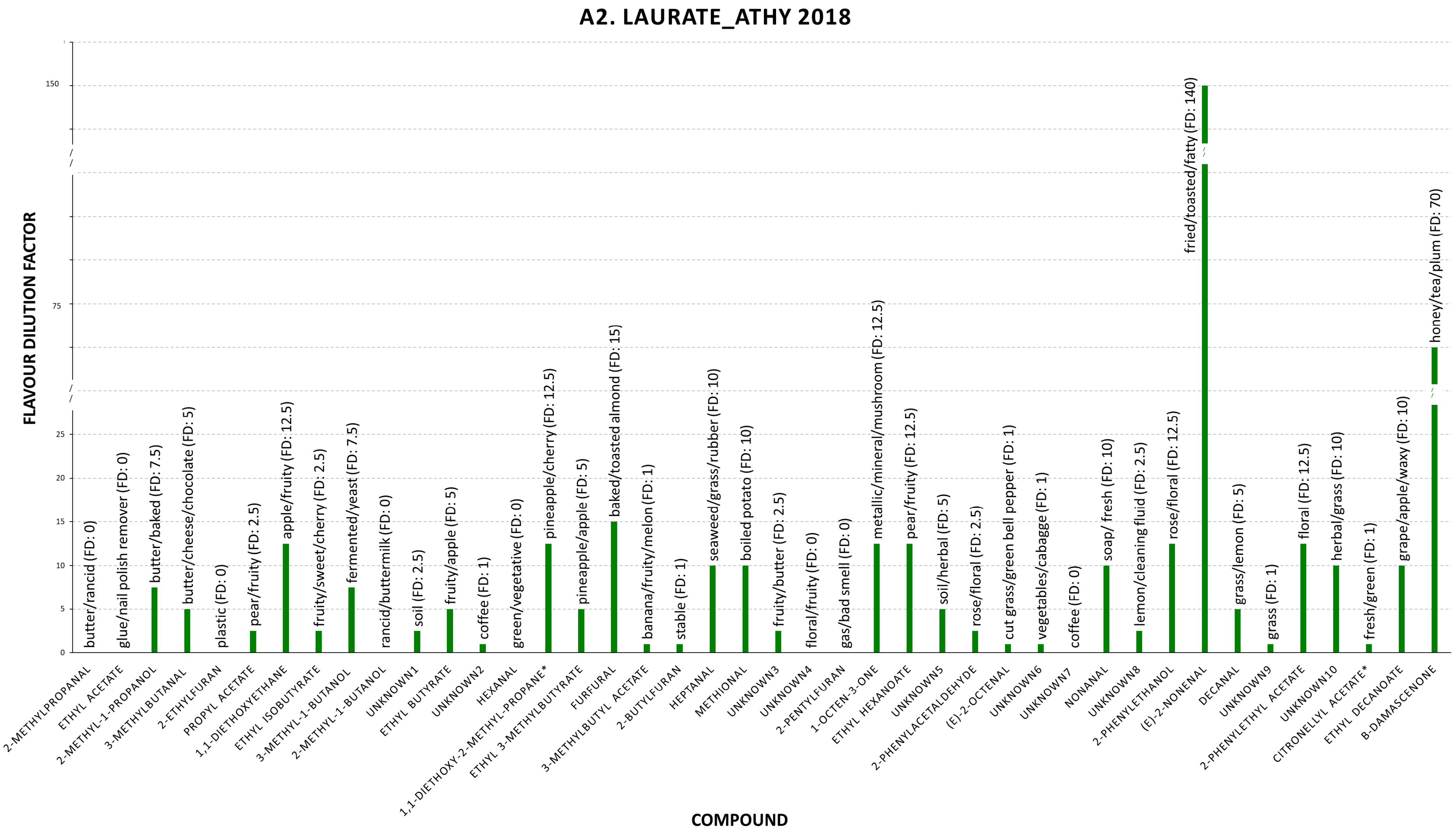

A2. LAURATE_ATHY 2018
FLAVOUR DILUTION FACTOR
COMPOUND
150
75
25
20
15
10
5
0
butter/rancid (FD: 0)
glue/nail polish remover (FD: 0)
butter/baked (FD: 7.5)
butter/cheese/chocolate (FD: 5)
plastic (FD: 0)
pear/fruity (FD: 2.5)
apple/fruity (FD: 12.5)
fruity/sweet/cherry (FD: 2.5)
fermented/yeast (FD: 7.5)
rancid/buttermilk (FD: 0)
soil (FD: 2.5)
fruity/apple (FD: 5)
coffee (FD: 1)
green/vegetative (FD: 0)
pineapple/cherry (FD: 12.5)
pineapple/apple (FD: 5)
baked/toasted almond (FD: 15)
banana/fruity/melon (FD: 1)
stable (FD: 1)
seaweed/grass/rubber (FD: 10)
boiled potato (FD: 10)
fruity/butter (FD: 2.5)
floral/fruity (FD: 0)
gas/bad smell (FD: 0)
metallic/mineral/mushroom (FD: 12.5)
pear/fruity (FD: 12.5)
soil/herbal (FD: 5)
rose/floral (FD: 2.5)
cut grass/green bell pepper (FD: 1)
vegetables/cabagge (FD: 1)
coffee (FD: 0)
soap/ fresh (FD: 10)
lemon/cleaning fluid (FD: 2.5)
rose/floral (FD: 12.5)
fried/toasted/fatty (FD: 140)
grass/lemon (FD: 5)
grass (FD: 1)
floral (FD: 12.5)
herbal/grass (FD: 10)
fresh/green (FD: 1)
grape/apple/waxy (FD: 10)
honey/tea/plum (FD: 70)
2-METHYLPROPANAL
ETHYL ACETATE
2-METHYL-1-PROPANOL
3-METHYLBUTANAL
2-ETHYLFURAN
PROPYL ACETATE
1,1-DIETHOXYETHANE
ETHYL ISOBUTYRATE
3-METHYL-1-BUTANOL
2-METHYL-1-BUTANOL
UNKNOWN1
ETHYL BUTYRATE
UNKNOWN2
HEXANAL
1,1-DIETHOXY-2-METHYL-PROPANE*
ETHYL 3-METHYLBUTYRATE
FURFURAL
3-METHYLBUTYL ACETATE
2-BUTYLFURAN
HEPTANAL
METHIONAL
UNKNOWN3
UNKNOWN4
2-PENTYLFURAN
1-OCTEN-3-ONE
ETHYL HEXANOATE
UNKNOWN5
2-PHENYLACETALDEHYDE
(E)-2-OCTENAL
UNKNOWN6
UNKNOWN7
NONANAL
UNKNOWN8
2-PHENYLETHANOL
(E)-2-NONENAL
DECANAL
UNKNOWN9
2-PHENYLETHYL ACETATE
UNKNOWN10
CITRONELLYL ACETATE*
ETHYL DECANOATE
B-DAMASCENONE

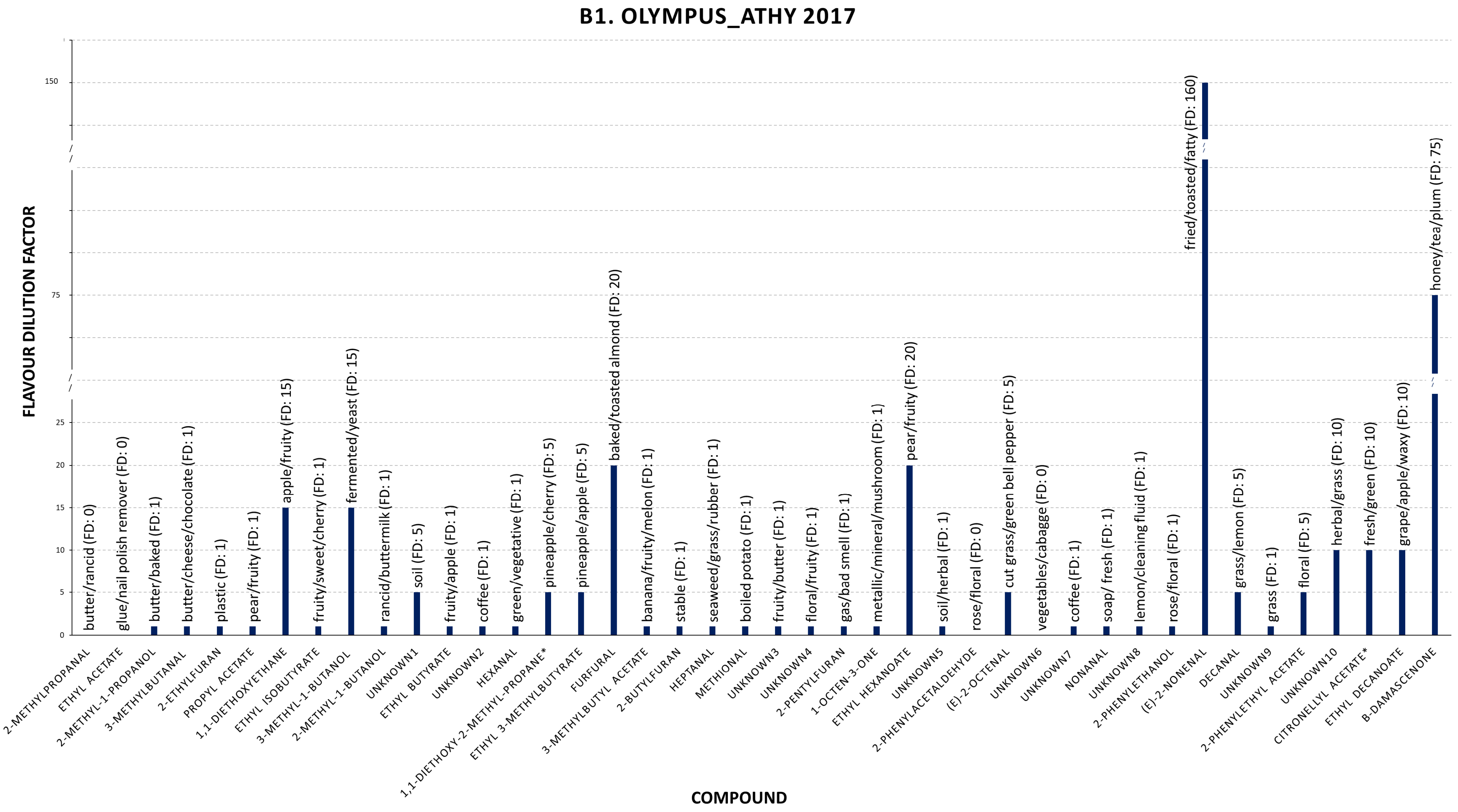
B1. OLYMPUS_ATHY 2017
FLAVOUR DILUTION FACTOR
150
75
25
20
15
10
5
0
butter/rancid (FD: 0)
glue/nail polish remover (FD: 0)
butter/baked (FD: 1)
butter/cheese/chocolate (FD: 1)
plastic (FD: 1)
pear/fruity (FD: 1)
apple/fruity (FD: 15)
fruity/sweet/cherry (FD: 1)
fermented/yeast (FD: 15)
rancid/buttermilk (FD: 1)
soil (FD: 5)
fruity/apple (FD: 1)
coffee (FD: 1)
green/vegetative (FD: 1)
pineapple/cherry (FD: 5)
pineapple/apple (FD: 5)
baked/toasted almond (FD: 20)
banana/fruity/melon (FD: 1)
stable (FD: 1)
seaweed/grass/rubber (FD: 1)
boiled potato (FD: 1)
fruity/butter (FD: 1)
floral/fruity (FD: 1)
gas/bad smell (FD: 1)
metallic/mineral/mushroom (FD: 1)
pear/fruity (FD: 20)
soil/herbal (FD: 1)
rose/floral (FD: 0)
cut grass/green bell pepper (FD: 5)
vegetables/cabagge (FD: 0)
coffee (FD: 1)
soap/ fresh (FD: 1)
lemon/cleaning fluid (FD: 1)
rose/floral (FD: 1)
fried/toasted/fatty (FD: 160)
grass/lemon (FD: 5)
grass (FD: 1)
floral (FD: 5)
herbal/grass (FD: 10)
fresh/green (FD: 10)
grape/apple/waxy (FD: 10)
honey/tea/plum (FD: 75)
2-METHYLPROPANAL
ETHYL ACETATE
2-METHYL-1-PROPANOL
3-METHYLBUTANAL
2-ETHYLFURAN
PROPYL ACETATE
1,1-DIETHOXYETHANE
ETHYL ISOBUTYRATE
3-METHYL-1-BUTANOL
2-METHYL-1-BUTANOL
UNKNOWN1
ETHYL BUTYRATE
UNKNOWN2
HEXANAL
1,1-DIETHOXY-2-METHYL-PROPANE*
ETHYL 3-METHYLBUTYRATE
FURFURAL
3-METHYLBUTYL ACETATE
2-BUTYLFURAN
HEPTANAL
METHIONAL
UNKNOWN3
UNKNOWN4
2-PENTYLFURAN
1-OCTEN-3-ONE
ETHYL HEXANOATE
UNKNOWN5
2-PHENYLACETALDEHYDE
(E)-2-OCTENAL
UNKNOWN6
UNKNOWN7
NONANAL
UNKNOWN8
2-PHENYLETHANOL
(E)-2-NONENAL
DECANAL
UNKNOWN9
2-PHENYLETHYL ACETATE
UNKNOWN10
CITRONELLYL ACETATE*
ETHYL DECANOATE
B-DAMASCENONE
COMPOUND

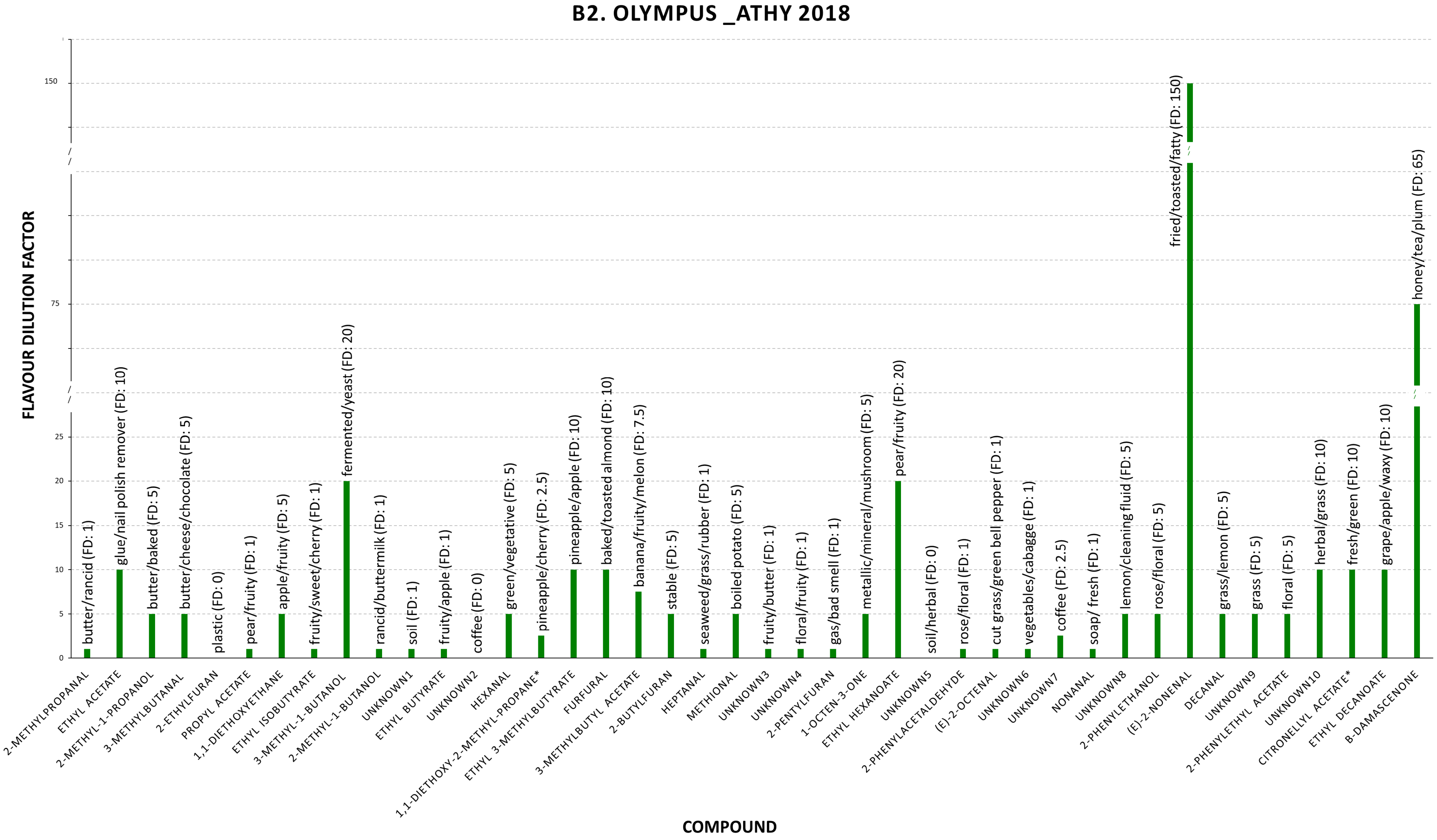
B2. OLYMPUS _ATHY 2018
FLAVOUR DILUTION FACTOR
COMPOUND
0
5
10
15
20
25
75
150
butter/rancid (FD: 1)
glue/nail polish remover (FD: 10)
butter/baked (FD: 5)
butter/cheese/chocolate (FD: 5)
plastic (FD: 0)
pear/fruity (FD: 1)
apple/fruity (FD: 5)
fruity/sweet/cherry (FD: 1)
fermented/yeast (FD: 20)
rancid/buttermilk (FD: 1)
soil (FD: 1)
fruity/apple (FD: 1)
coffee (FD: 0)
green/vegetative (FD: 5)
pineapple/cherry (FD: 2.5)
pineapple/apple (FD: 10)
baked/toasted almond (FD: 10)
banana/fruity/melon (FD: 7.5)
stable (FD: 5)
seaweed/grass/rubber (FD: 1)
boiled potato (FD: 5)
fruity/butter (FD: 1)
floral/fruity (FD: 1)
gas/bad smell (FD: 1)
metallic/mineral/mushroom (FD: 5)
pear/fruity (FD: 20)
soil/herbal (FD: 0)
rose/floral (FD: 1)
cut grass/green bell pepper (FD: 1)
vegetables/cabagge (FD: 1)
coffee (FD: 2.5)
soap/ fresh (FD: 1)
lemon/cleaning fluid (FD: 5)
rose/floral (FD: 5)
fried/toasted/fatty (FD: 150)
grass/lemon (FD: 5)
grass (FD: 5)
floral (FD: 5)
herbal/grass (FD: 10)
fresh/green (FD: 10)
grape/apple/waxy (FD: 10)
honey/tea/plum (FD: 65)
2-METHYLPROPANAL
ETHYL ACETATE
2-METHYL-1-PROPANOL
3-METHYLBUTANAL
2-ETHYLFURAN
PROPYL ACETATE
1,1-DIETHOXYETHANE
ETHYL ISOBUTYRATE
3-METHYL-1-BUTANOL
2-METHYL-1-BUTANOL
UNKNOWN1
ETHYL BUTYRATE
UNKNOWN2
HEXANAL
1,1-DIETHOXY-2-METHYL-PROPANE*
ETHYL 3-METHYLBUTYRATE
FURFURAL
3-METHYLBUTYL ACETATE
2-BUTYLFURAN
HEPTANAL
METHIONAL
UNKNOWN3
UNKNOWN4
2-PENTYLFURAN
1-OCTEN-3-ONE
ETHYL HEXANOATE
UNKNOWN5
2-PHENYLACETALDEHYDE
(E)-2-OCTENAL
UNKNOWN6
UNKNOWN7
NONANAL
UNKNOWN8
2-PHENYLETHANOL
(E)-2-NONENAL
DECANAL
UNKNOWN9
2-PHENYLETHYL ACETATE
UNKNOWN10
CITRONELLYL ACETATE*
ETHYL DECANOATE
B-DAMASCENONE

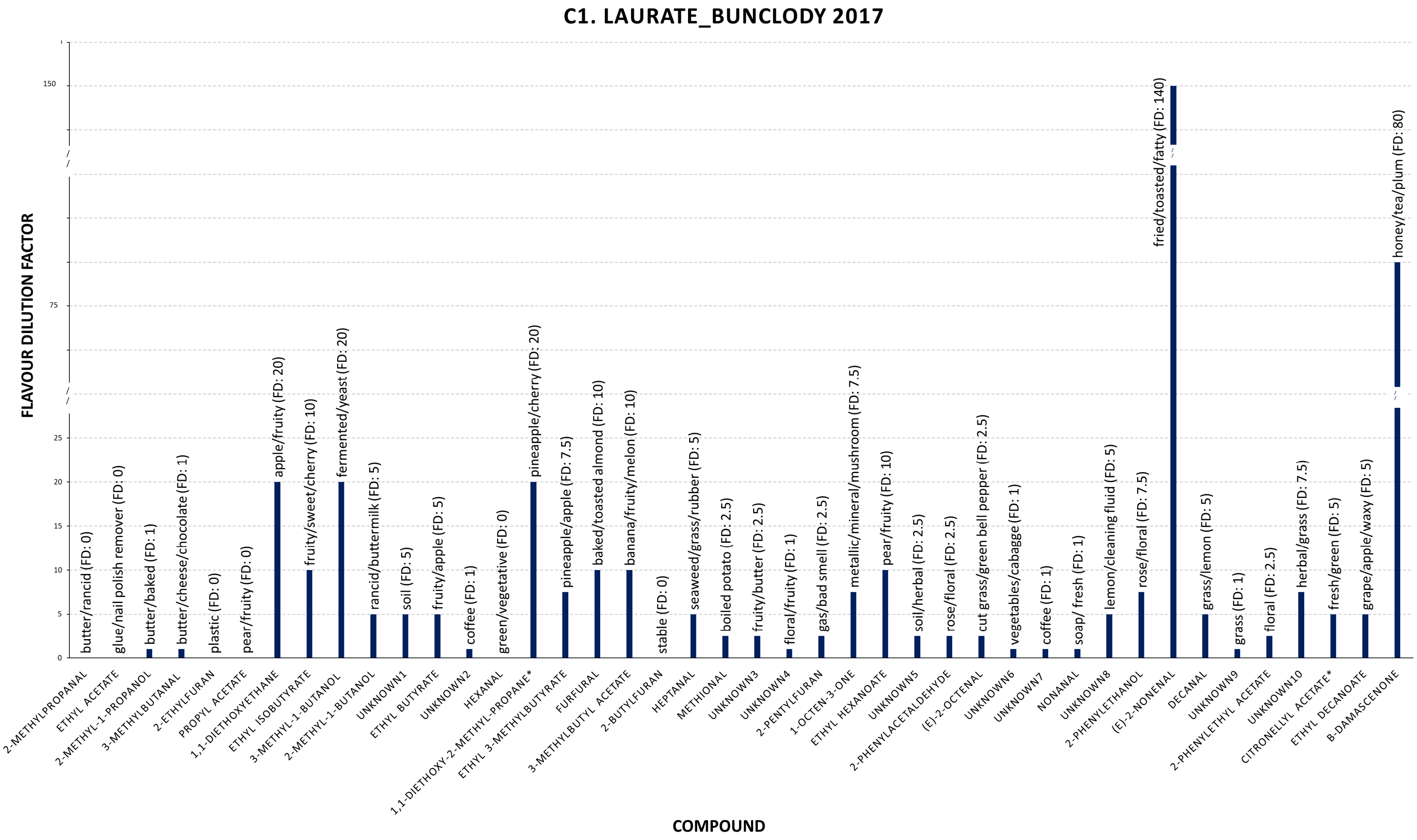
C1. LAURATE_BUNCLODY 2017
FLAVOUR DILUTION FACTOR
0
5
10
15
20
25
75
150
COMPOUND
butter/rancid (FD: 0)
glue/nail polish remover (FD: 0)
butter/baked (FD: 1)
butter/cheese/chocolate (FD: 1)
plastic (FD: 0)
pear/fruity (FD: 0)
apple/fruity (FD: 20)
fruity/sweet/cherry (FD: 10)
fermented/yeast (FD: 20)
rancid/buttermilk (FD: 5)
soil (FD: 5)
fruity/apple (FD: 5)
coffee (FD: 1)
green/vegetative (FD: 0)
pineapple/cherry (FD: 20)
pineapple/apple (FD: 7.5)
baked/toasted almond (FD: 10)
banana/fruity/melon (FD: 10)
stable (FD: 0)
seaweed/grass/rubber (FD: 5)
boiled potato (FD: 2.5)
fruity/butter (FD: 2.5)
floral/fruity (FD: 1)
gas/bad smell (FD: 2.5)
metallic/mineral/mushroom (FD: 7.5)
pear/fruity (FD: 10)
soil/herbal (FD: 2.5)
rose/floral (FD: 2.5)
cut grass/green bell pepper (FD: 2.5)
vegetables/cabagge (FD: 1)
coffee (FD: 1)
soap/ fresh (FD: 1)
lemon/cleaning fluid (FD: 5)
rose/floral (FD: 7.5)
fried/toasted/fatty (FD: 140)
grass/lemon (FD: 5)
grass (FD: 1)
floral (FD: 2.5)
herbal/grass (FD: 7.5)
fresh/green (FD: 5)
grape/apple/waxy (FD: 5)
honey/tea/plum (FD: 80)
2-METHYLPROPANAL
ETHYL ACETATE
2-METHYL-1-PROPANOL
3-METHYLBUTANAL
2-ETHYLFURAN
PROPYL ACETATE
1,1-DIETHOXYETHANE
ETHYL ISOBUTYRATE
3-METHYL-1-BUTANOL
2-METHYL-1-BUTANOL
UNKNOWN1
ETHYL BUTYRATE
UNKNOWN2
HEXANAL
1,1-DIETHOXY-2-METHYL-PROPANE*
ETHYL 3-METHYLBUTYRATE
FURFURAL
3-METHYLBUTYL ACETATE
2-BUTYLFURAN
HEPTANAL
METHIONAL
UNKNOWN3
UNKNOWN4
2-PENTYLFURAN
1-OCTEN-3-ONE
ETHYL HEXANOATE
UNKNOWN5
2-PHENYLACETALDEHYDE
(E)-2-OCTENAL
UNKNOWN6
UNKNOWN7
NONANAL
UNKNOWN8
2-PHENYLETHANOL
(E)-2-NONENAL
DECANAL
UNKNOWN9
2-PHENYLETHYL ACETATE
UNKNOWN10
CITRONELLYL ACETATE*
ETHYL DECANOATE
B-DAMASCENONE

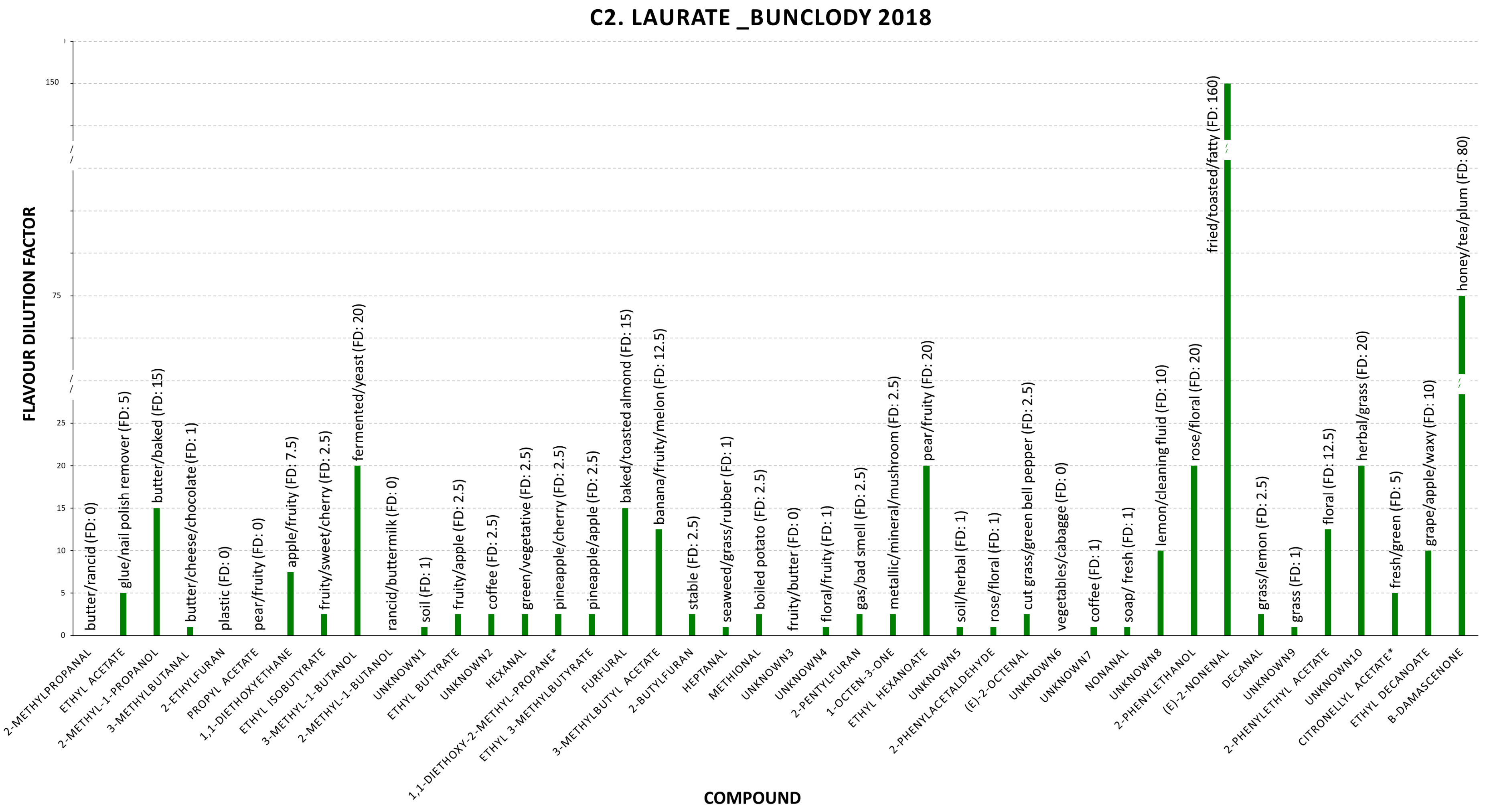

C2. LAURATE _BUNCLODY 2018
FLAVOUR DILUTION FACTOR
150
75
25
20
15
10
5
0
butter/rancid (FD: 0)
glue/nail polish remover (FD: 5)
butter/baked (FD: 15)
butter/cheese/chocolate (FD: 1)
plastic (FD: 0)
pear/fruity (FD: 0)
apple/fruity (FD: 7.5)
fruity/sweet/cherry (FD: 2.5)
fermented/yeast (FD: 20)
rancid/buttermilk (FD: 0)
soil (FD: 1)
fruity/apple (FD: 2.5)
coffee (FD: 2.5)
green/vegetative (FD: 2.5)
pineapple/cherry (FD: 2.5)
pineapple/apple (FD: 2.5)
baked/toasted almond (FD: 15)
banana/fruity/melon (FD: 12.5)
stable (FD: 2.5)
seaweed/grass/rubber (FD: 1)
boiled potato (FD: 2.5)
fruity/butter (FD: 0)
floral/fruity (FD: 1)
gas/bad smell (FD: 2.5)
metallic/mineral/mushroom (FD: 2.5)
pear/fruity (FD: 20)
soil/herbal (FD: 1)
rose/floral (FD: 1)
cut grass/green bell pepper (FD: 2.5)
vegetables/cabagge (FD: 0)
coffee (FD: 1)
soap/ fresh (FD: 1)
lemon/cleaning fluid (FD: 10)
rose/floral (FD: 20)
fried/toasted/fatty (FD: 160)
grass/lemon (FD: 2.5)
grass (FD: 1)
floral (FD: 12.5)
herbal/grass (FD: 20)
fresh/green (FD: 5)
grape/apple/waxy (FD: 10)
honey/tea/plum (FD: 80)
2-METHYLPROPANAL
ETHYL ACETATE
2-METHYL-1-PROPANOL
3-METHYLBUTANAL
2-ETHYLFURAN
PROPYL ACETATE
1,1-DIETHOXYETHANE
ETHYL ISOBUTYRATE
3-METHYL-1-BUTANOL
2-METHYL-1-BUTANOL
UNKNOWN1
ETHYL BUTYRATE
UNKNOWN2
HEXANAL
1,1-DIETHOXY-2-METHYL-PROPANE*
ETHYL 3-METHYLBUTYRATE
FURFURAL
3-METHYLBUTYL ACETATE
2-BUTYLFURAN
HEPTANAL
METHIONAL
UNKNOWN3
UNKNOWN4
2-PENTYLFURAN
1-OCTEN-3-ONE
ETHYL HEXANOATE
UNKNOWN5
2-PHENYLACETALDEHYDE
(E)-2-OCTENAL
UNKNOWN6
UNKNOWN7
NONANAL
UNKNOWN8
2-PHENYLETHANOL
(E)-2-NONENAL
DECANAL
UNKNOWN9
2-PHENYLETHYL ACETATE
UNKNOWN10
CITRONELLYL ACETATE*
ETHYL DECANOATE
B-DAMASCENONE
COMPOUND

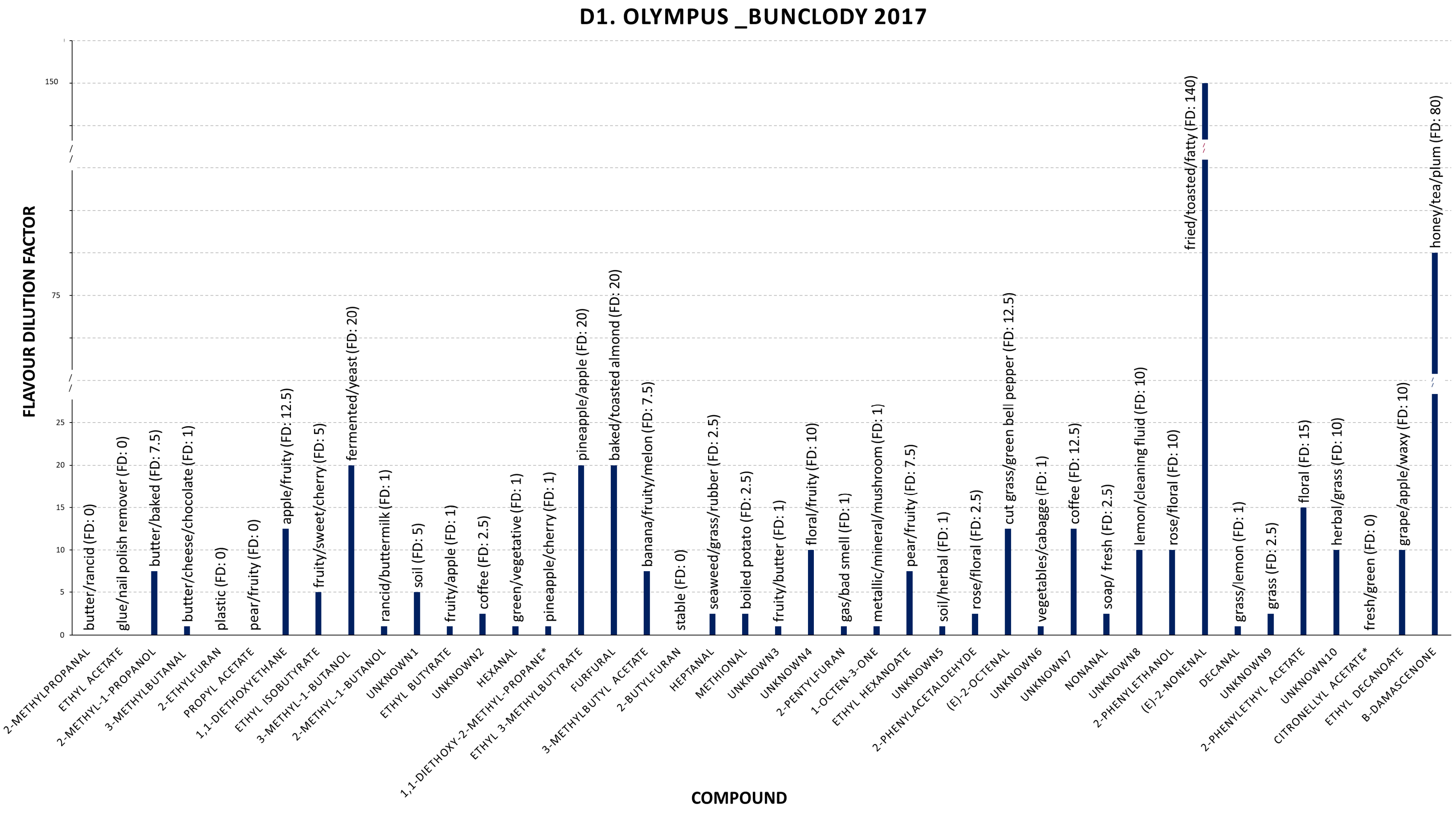
D1. OLYMPUS _BUNCLODY 2017
FLAVOUR DILUTION FACTOR
COMPOUND
0
5
10
15
20
25
75
150
butter/rancid (FD: 0)
glue/nail polish remover (FD: 0)
butter/baked (FD: 7.5)
butter/cheese/chocolate (FD: 1)
plastic (FD: 0)
pear/fruity (FD: 0)
apple/fruity (FD: 12.5)
fruity/sweet/cherry (FD: 5)
fermented/yeast (FD: 20)
rancid/buttermilk (FD: 1)
soil (FD: 5)
fruity/apple (FD: 1)
coffee (FD: 2.5)
green/vegetative (FD: 1)
pineapple/cherry (FD: 1)
pineapple/apple (FD: 20)
baked/toasted almond (FD: 20)
banana/fruity/melon (FD: 7.5)
stable (FD: 0)
seaweed/grass/rubber (FD: 2.5)
boiled potato (FD: 2.5)
fruity/butter (FD: 1)
floral/fruity (FD: 10)
gas/bad smell (FD: 1)
metallic/mineral/mushroom (FD: 1)
pear/fruity (FD: 7.5)
soil/herbal (FD: 1)
rose/floral (FD: 2.5)
cut grass/green bell pepper (FD: 12.5)
vegetables/cabagge (FD: 1)
coffee (FD: 12.5)
soap/ fresh (FD: 2.5)
lemon/cleaning fluid (FD: 10)
rose/floral (FD: 10)
fried/toasted/fatty (FD: 140)
grass/lemon (FD: 1)
grass (FD: 2.5)
floral (FD: 15)
herbal/grass (FD: 10)
fresh/green (FD: 0)
grape/apple/waxy (FD: 10)
honey/tea/plum (FD: 80)
2-METHYLPROPANAL
ETHYL ACETATE
2-METHYL-1-PROPANOL
3-METHYLBUTANAL
2-ETHYLFURAN
PROPYL ACETATE
1,1-DIETHOXYETHANE
ETHYL ISOBUTYRATE
3-METHYL-1-BUTANOL
2-METHYL-1-BUTANOL
UNKNOWN1
ETHYL BUTYRATE
UNKNOWN2
HEXANAL
1,1-DIETHOXY-2-METHYL-PROPANE*
ETHYL 3-METHYLBUTYRATE
FURFURAL
3-METHYLBUTYL ACETATE
2-BUTYLFURAN
HEPTANAL
METHIONAL
UNKNOWN3
UNKNOWN4
2-PENTYLFURAN
1-OCTEN-3-ONE
ETHYL HEXANOATE
UNKNOWN5
2-PHENYLACETALDEHYDE
(E)-2-OCTENAL
UNKNOWN6
UNKNOWN7
NONANAL
UNKNOWN8
2-PHENYLETHANOL
(E)-2-NONENAL
DECANAL
UNKNOWN9
2-PHENYLETHYL ACETATE
UNKNOWN10
CITRONELLYL ACETATE*
ETHYL DECANOATE
B-DAMASCENONE

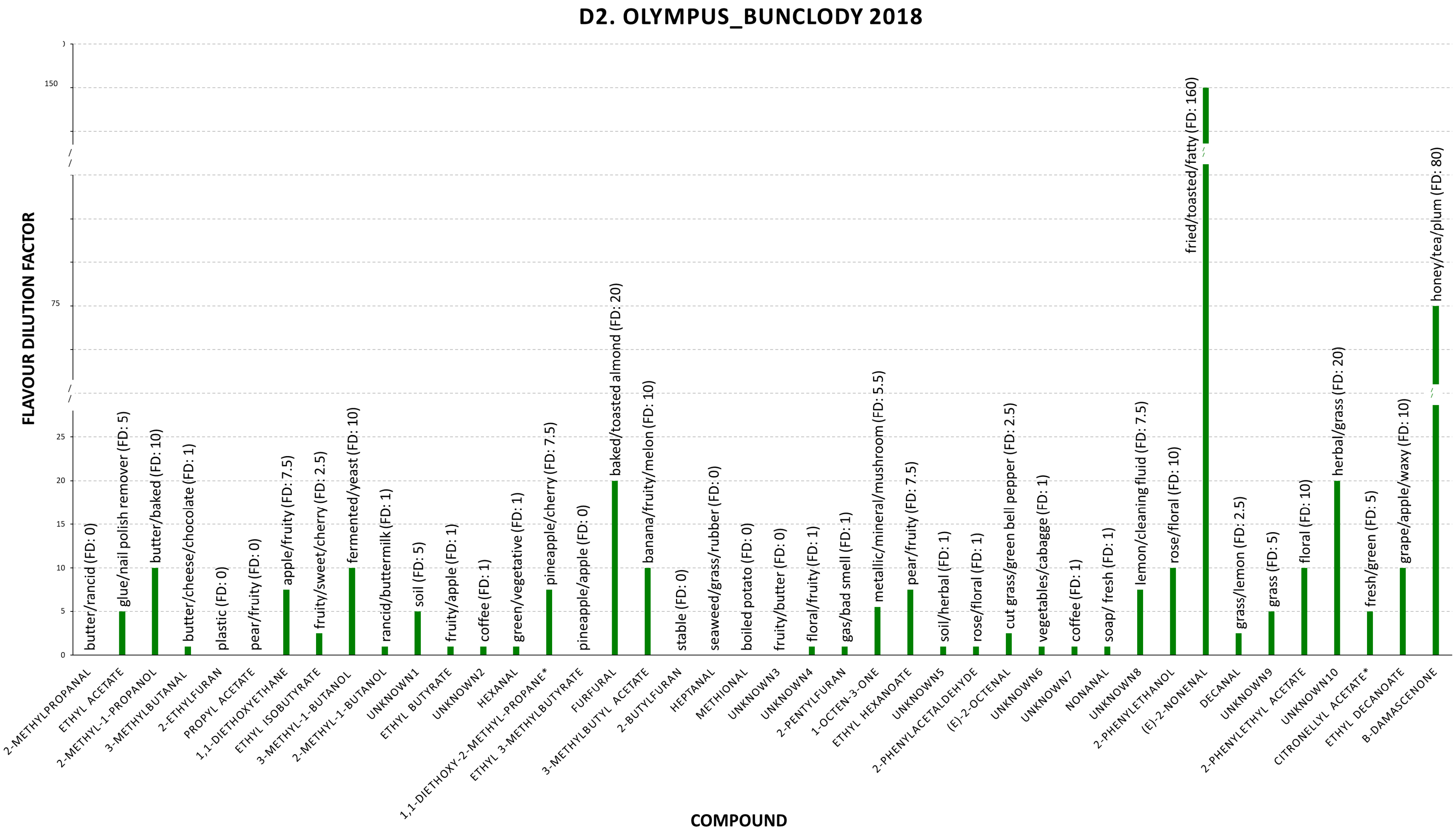

D2. OLYMPUS_BUNCLODY 2018
FLAVOUR DILUTION FACTOR
COMPOUND
2-METHYLPROPANAL butter/rancid (FD: 0)
ETHYL ACETATE glue/nail polish remover (FD: 5)
2-METHYL-1-PROPANOL butter/baked (FD: 10)
3-METHYLBUTANAL butter/cheese/chocolate (FD: 1)
2-ETHYLFURAN plastic (FD: 0)
PROPYL ACETATE pear/fruity (FD: 0)
1,1-DIETHOXYETHANE apple/fruity (FD: 7.5)
ETHYL ISOBUTYRATE fruity/sweet/cherry (FD: 2.5)
3-METHYL-1-BUTANOL fermented/yeast (FD: 10)
2-METHYL-1-BUTANOL rancid/buttermilk (FD: 1)
UNKNOWN1 soil (FD: 5)
ETHYL BUTYRATE fruity/apple (FD: 1)
UNKNOWN2 coffee (FD: 1)
HEXANAL green/vegetative (FD: 1)
1,1-DIETHOXY-2-METHYL-PROPANE* pineapple/cherry (FD: 7.5)
ETHYL 3-METHYLBUTYRATE pineapple/apple (FD: 0)
FURFURAL baked/toasted almond (FD: 20)
3-METHYLBUTYL ACETATE banana/fruity/melon (FD: 10)
2-BUTYLFURAN stable (FD: 0)
HEPTANAL seaweed/grass/rubber (FD: 0)
METHIONAL boiled potato (FD: 0)
UNKNOWN3 fruity/butter (FD: 0)
UNKNOWN4 floral/fruity (FD: 1)
2-PENTYLFURAN gas/bad smell (FD: 1)
1-OCTEN-3-ONE metallic/mineral/mushroom (FD: 5.5)
ETHYL HEXANOATE pear/fruity (FD: 7.5)
UNKNOWN5 soil/herbal (FD: 1)
2-PHENYLACETALDEHYDE rose/floral (FD: 1)
(E)-2-OCTENAL cut grass/green bell pepper (FD: 2.5)
UNKNOWN6 vegetables/cabagge (FD: 1)
UNKNOWN7 coffee (FD: 1)
NONANAL soap/ fresh (FD: 1)
UNKNOWN8 lemon/cleaning fluid (FD: 7.5)
2-PHENYLETHANOL rose/floral (FD: 10)
(E)-2-NONENAL fried/toasted/fatty (FD: 160)
DECANAL grass/lemon (FD: 2.5)
UNKNOWN9 grass (FD: 5)
2-PHENYLETHYL ACETATE floral (FD: 10)
UNKNOWN10 herbal/grass (FD: 20)
CITRONELLYL ACETATE* fresh/green (FD: 5)
ETHYL DECANOATE grape/apple/waxy (FD: 10)
Β-DAMASCENONE honey/tea/plum (FD: 80)
0
5
10
15
20
25
75
150

Supplementary Table 1. Average soil nutrients[1] from Athy, Co. Kildare and Bunclody, Co. Wexford across 2017 and 2018 seasons.

Site	pH	OM	Ca	P	K	Mg	Na	Mn	B	Cu	Zn	Mo
Athy	7.8	7.5	1221	8.2	168	46.9	24.2	0.51	1.11	258	743	35
Bunclody	6.6	7.7	369	5.9	157	16.1	25.6	0.46	1.26	238	451	18

[1] All nutrient values are reported in mg/L, except Cu, Zn, and Mo which are reported in µg/L
Results expressed at 95% confidence interval
OM: Organic matter

Supplementary Table 2. Average weather measurements from Athy, Co. Kildare and Bunclody, Co. Wexford across 2017 and 2018 seasons.

Location	Season	Month	Temperature (°C)	Rainfall (mm)	Humidity (%)	Wind Speed (mph)	Wind Chill (°C)	Dew Point (°C)	Barometer (inHg)	Heat Index (°C)	THW Index (°C)	Heating Degree Days	Cooling Degree Days
Athy	2017	April	15.7	38.1	73.4	2.7	15.6	8.4	29.8	15.4	15.4	42.4	6.7
		May	15.1	18.1	82.6	2.4	15.1	10.8	30.1	15.1	15.1	47.7	3.9
		June	10.0	15.1	80.1	3.6	9.5	6.4	30.0	9.8	9.3	112.6	0.1
		July	14.8	7.3	76.8	3.0	14.6	9.6	30.2	14.7	14.6	54.6	6.9
		August	16.5	30.4	78.4	2.9	16.4	12.3	30.0	16.5	16.4	35.4	10.6
	2018	April	11.5	27.9	79.9	7.9	10.0	8.0	29.8	11.4	9.8	80.3	1.5
		May	10.8	5.1	79.2	5.5	9.8	7.1	30.0	10.6	9.6	60.7	0.1
		June	12.5	12.7	84.0	4.9	11.9	9.7	29.8	12.5	11.8	44.0	2.6
		July	16.0	10.2	83.6	4.5	15.8	13.1	29.9	16.2	15.9	23.0	7.1
		August	15.4	12.7	85.6	5.9	14.9	12.9	29.8	15.5	15.0	22.9	3.8
Bunclody	2017	April	9.4	51.9	84.6	6.0	8.1	6.8	29.9	9.3	8.0	182.4	0.0
		May	13.4	8.6	79.0	3.3	13.2	9.7	30.2	13.3	13.1	102.3	2.8
		June	15.9	20.4	80.5	3.1	15.8	12.4	30.1	15.9	15.8	160.2	11.3
		July	15.8	97.0	77.9	5.0	15.5	11.7	29.9	15.8	15.4	64.2	13.2

	August	14.9	144.0	82.4	4.8	14.6	11.8	30.0	14.9	14.6	109.5	4.3
2018	April	11.1	45.7	85.1	5.6	10.1	8.5	29.9	11.1	10.1	98.4	0.8
	May	11.1	10.2	82.1	5.6	10.1	8.0	30.0	11.0	10.0	68.3	0.6
	June	12.9	12.7	85.3	4.9	12.3	10.4	29.9	13.0	12.3	48.9	3.2
	July	16.4	27.9	84.8	4.8	16.1	13.6	30.0	16.6	16.3	24.8	8.9
	August	15.7	20.3	86.3	5.9	15.2	13.4	29.8	15.9	15.4	39.8	5.0

Supplementary Table 3: Odour description of the pens from the Sniffin' Sticks (Test Blue Kit).

Stick Number	Odour				Correct answer
**1**	Orange	Banana	Fish	Coffee	Orange
**2**	Honey	Strawberry	Leather	Bread	Leather
**3**	Thyme	Fish	Cinnamon	Banana	Cinnamon
**4**	Mustard	Peppermint	Sweat	Bread	Peppermint
**5**	Cheese	Soap	Banana	Rose	Banana
**6**	Vinegar	Banana	Fish	Mustard	Banana
**7**	Liquorice	Vanilla	Cheese	Apple	Liquorice
**8**	Smoke	French Fries	Paint Thinner	Onion	Paint Thinner
**9**	Garbage	Smoke	Garlic	Cheese	Garlic
**10**	Coffee	Apple	Petrol	Grass	Coffee
**11**	Garlic	Chocolate	Apple	Orange	Apple
**12**	Onion	Cloves	Meat	Vinegar	Cloves
**13**	Tomato	Shrimps	Pineapple	Smoke	Pineapple
**14**	Mint	Rose	Apple	Mushrooms	Rose
**15**	Onion	Coffee	Anise	Meat	Anise
**16**	Tobacco	Strawberry	Basil	Fish	Fish

Supplementary Table 4: Odours detected in new-make spirits and used for the GCO analysis

RT	Attribute	Odour Intensity	RT	Attribute	Odour Intensity
3.20	butter/rancid		13.80	fruity/butter	
4.30	glue/nail polish remover		13.96	floral/fruity	
5.15	butter/baked		14.36	gas/bad smell	
5.33	butter/cheese/chocolate		14.70	metallic/mineral/mushroom	
5.94	plastic		14.89	pear/fruity	
6.71	pear/fruity		16.27	soil/herbal	
6.81	apple/fruity		17.16	rose/floral	
7.90	fruity/sweet/cherry		17.28	cut grass/green bell pepper	
8.08	fermented/yeast		17.35	vegetables/cabagge	
8.15	rancid/buttermilk		17.73	coffee	
8.25	soil		18.07	soap/ fresh	
9.16	fruity/apple		19.22	lemon/cleaning fluid	
9.22	coffee		19.30	rose/floral	
9.50	green/vegetative		19.80	fried/toasted/fatty	
10.55	pineapple/cherry		20.20	grass/lemon	
10.70	pineapple/apple		20.89	grass	
11.20	baked/toasted almond		21.30	floral	
11.50	banana/fruity/melon		21.41	herbal/grass	
12.20	stable		22.26	fresh/green	
12.50	seaweed/grass/rubber		22.70	grape/apple/waxy	
13.31	boiled potato		23.03	honey/tea/plum	

Supplementary Table 5: Standard mix solution in 20% of ethanol in water

Compound	Cas Number	Concentration (mg/L)
2-Ethylfuran	3208-16-0	1
1-Octen-3-one	4312-99-6	0.6
2-Pentylfuran	3777-69-3	1
(E)-2-Nonenal	18829-56-6	0.05
3-Methyl-1-butanol (Isoamyl alcohol)	123-51-3	200
3-Methylbutanal (Isovaleraldehyde)	590-86-3	4
β-Damascenone	23726-93-4	1.5
2-Phenylethanol	60-12-8	10
2-Phenylethyl acetate	103-45-7	10
Ethyl hexanoate	123-66-0	2
3-Methylbutyl acetate (Isoamyl acetate)	123-92-2	3
Ethyl isobutyrate	97-62-1	10
1,1-Diethoxyethane	105-57-7	100
Ethyl 3-methylbutyrate (Ethyl Isovalerate)	108-64-5	9
Heptanal	111-71-7	3
Ethyl decanoate	110-38-3	10
Methional	3268-49-3	5
(E)-2-Octenal	2548-87-0	3
Hexanal	66-25-1	16
